# Exosomes released by environmental pollutant-stimulated Keratinocytes/PBMCs can trigger psoriatic inflammation in recipient cells via the AhR signaling pathway

**DOI:** 10.3389/fmolb.2023.1324692

**Published:** 2024-01-15

**Authors:** Hye Ran Kim, So Yeon Lee, Ga Eun You, Chun Wook Park, Hye One Kim, Bo Young Chung

**Affiliations:** ^1^ Department of Dermatology, Kangnam Sacred Heart Hospital, Hallym University College of Medicine, Seoul, Republic of Korea; ^2^ Research and Development Institute, Biosolution, Seoul, Republic of Korea

**Keywords:** exosomes, benzo[a]pyrene, aryl hydrocarbon receptor, psoriasis, 2,3,7,8-tetrachlorodibenzo-p-dioxin (TCDD)

## Abstract

**Introduction:** Exosomes, pivotal in intercellular communication during skin disease pathogenesis, have garnered substantial attention. However, the impact of environmental pollutants, such as benzo[a]pyrene (BaP) and 2, 3, 7, 8-tetrachlorodibenzo-p-dioxin (TCDD), on exosome release amid inflammatory skin diseases remains unexplored. This study addresses this gap by examining the influence of BaP and TCDD on exosome function, specifically focusing on immune-related pathway alterations in normal recipient keratinocytes and peripheral blood mononuclear cells (PBMCs).

**Methods:** HaCaT cells were treated with exosomes from BaP- or TCDD-treated keratinocytes. Proinflammatory cytokines and chemokines, including TNF-α, IL-1β, IL-6, IL-8, CXCL1, and CXCL5, were assessed. The involvement of the p65NF-κB/p38MAPK/ERK signaling pathway in recipient keratinocytes was investigated. Aryl hydrocarbon receptor (AhR) silencing was employed to elucidate its role in mediating the proinflammatory response induced by exosomes from BaP- or TCDD-treated keratinocytes.

**Results and discussion:** Treatment with exosomes from BaP- or TCDD-treated keratinocytes induced a significant increase in proinflammatory cytokines and chemokines in HaCaT cells. The upregulation implicated the p65NF-κB/p38MAPK/ERK signaling pathway. AhR silencing attenuated this response, suggesting a role for AhR in mediating this response. In PBMCs from healthy controls, exosomes from BaP-stimulated PBMCs of psoriatic patients led to increased expression of proinflammatory cytokines and modulation of Th1/Th17 cell distribution via AhR activation. These findings unveil a novel dimension in the interplay between environmental xenobiotic agents (BaP and TCDD) and exosomal functions. The study establishes their influence on psoriatic inflammatory responses, shedding light on the underlying mechanisms mediated through the AhR signaling pathway in recipient keratinocytes and PBMCs.

## 1 Introduction

Benzo[a]pyrene (BaP), a polycyclic aromatic hydrocarbon and a ubiquitous environmental pollutant, is produced through the incomplete combustion of diesel exhaust, tobacco smoke, and industrial waste (Kamal et al., 2015). Humans are exposed to benzo[a]pyrene through smoking, which is known to exacerbate inflammatory skin diseases such as psoriasis (Fortes et al., 2005). The environmental chemical TCDD, belonging to the “polyhalogenated aromatic hydrocarbon” (PHAH) family, is identified as an endocrine-disrupting chemical (EDC) (Hotchkiss et al., 2008). It is commonly known as dioxin and is predominantly produced from waste incineration, metal production, fossil fuels, and wood combustion (Schecter and Gasiewicz, 2003). Due to its lipophilic nature, TCDD easily penetrates animal tissues and accumulates in the food chain, making animal-derived food the primary source of human exposure (Antignac et al., 2006).

BaP and TCDD are metabolized in humans via the aryl hydrocarbon receptor (AhR) signaling pathway. Xenobiotic compounds, such as BaP and TCDD, bind to AhR, leading to the synthesis of members of the cytochrome P450 enzyme family, including subfamily A, polypeptide 1 (CYP1A1) (Anttila et al., 1992; Reyes et al., 1992; Ko et al., 1996; Duan et al., 2001; Esser et al., 2013). BaP or TCDD have been suggested to play a role in regulating inflammation, in addition to their involvement in carcinogenesis (Mandal, 2005; Chen and Liao, 2006; Hong et al., 2016; Gutierrez-Vazquez and Quintana, 2018; Kim et al., 2020). While BaP and TCDD have been implicated in regulating inflammation and carcinogenesis, there is a paucity of research on their effects on inflammatory immune response, particularly on the skin.

Previous studies have demonstrated the roles of BaP and TCDD in inducing inflammatory reactions in healthy mouse models. Alalaiwe et al. (2020) showed that BaP application elicited cutaneous inflammation, such as epidermal proliferation and infiltration of immune cells, in the skin of normal mice. Rudyak et al. (2018) found that TCDD application resulted in an inflammatory reaction in the skin, resembling that of psoriasis or dermatitis, including hyperproliferation, parakeratosis, infiltration of immune cells, and increased expression of inflammation-related genes, in normal mice models. Kim et al. (2020), Kim et al. (2021) reported that TCDD aggravates inflammatory reactions in keratinocytes and psoriasis-like mice via AhR activation (Kim et al., 2020; Kim et al., 2021).

Intercellular communication plays an important role in skin pathogenesis. Complicated communication between keratinocytes and immune cells, such as lymphocytes and neutrophils, can induce complex responses in the skin environment through internal or external stimuli (Xiong et al., 2021). Exosomes, cell-derived vesicles with a diameter of 40–160 nm, contain proteins, lipids, DNA, and miRNAs depending on the properties of the parent cell (Kalluri and LeBleu, 2020). They act as intercellular transmitters, shuttling paracrine mediators to neighboring cells and influencing the biological properties of recipient cells. Exosomes play a pivotal role in various physiological functions, including proliferation, differentiation, and cell death. Additionally, they are implicated in the pathological processes underlying various diseases (Than et al., 2019). Recent evidence highlights the potential of exosomes as promising therapeutic targets and biomarkers (Xunian and Kalluri, 2020). However, few studies have elucidated the regulatory mechanisms of exosomes in inflammatory skin diseases such as psoriasis. While the impact of environmental pollutants on exosomes has been explored in neurodegenerative diseases, several cancers, and lung and liver diseases (Harischandra et al., 2017; van Meteren et al., 2019), nothing is available concerning their effects on skin diseases. Consequently, this study aims to investigate whether exosomes released by environmental pollutants, such as those from BaP- or TCDD-treated keratinocytes or PBMCs, modulate inflammatory reactions and immune responses in recipient cells.

## 2 Methods

### 2.1 Study participants, isolation of peripheral blood mononuclear cells, and BaP treatment

Blood samples were obtained from both patients diagnosed with psoriasis (*n* = 5) and healthy volunteers (*n* = 5), all of whom provided written informed consent to participate in this study. Approval for this study was granted by the Institutional Review Board of Hallym University Kangnam Sacred Heart Hospital (IRB No. 2022-03-022). Peripheral blood mononuclear cells (PBMCs) were isolated from the blood samples of both patients with psoriasis and healthy volunteers. A 1:1 ratio of human blood samples to Ficoll-Paque solution (1.077 g/mL; Amersham Biosciences, Uppsala, Sweden) was used, followed by centrifugation at 800 × g for 15 min. The cells from the interphase were subsequently washed three times with PBS containing 5 mM EDTA. PBMCs derived from patients with psoriasis were treated with Benzo[a]pyrene (BaP) at a concentration of 10 µM (Sigma‒Aldrich, St. Louis, MO, United States) for 24 h. Subsequently, exosomes were isolated from BaP-treated PBMCs of patients with psoriasis, as well as from non-treated PBMCs of patients with psoriasis. Additionally, PBMCs from healthy controls (HCs) were treated with 20 μg/mL exosomes for 24 h.

### 2.2 HaCaT cell culture and treatment

The HaCaT cell line, an immortalized human keratinocyte cell line (Welgene, Daegu, South Korea), was cultured in Dulbecco’s modified Eagle’s medium (DMEM) (Lonza, Walkersville, MD, United States) supplemented with 10% fetal bovine serum (Gibco; Thermo Fisher Scientific, Waltham, MA, United States) and 1% penicillin-streptomycin. Cultures were maintained at 37°C in a humidified CO_2_ incubator (5% CO_2_). HaCaT cells were treated with 10 nM 2,3,7,8-Tetrachlorodibenzo-p-dioxin (TCDD) (Sigma–Aldrich) or 0.5 µM Benzo [a]pyrene (BaP) (Sigma–Aldrich) for 24 h. Exosomes were then isolated from TCDD- or BaP-treated HaCaT cells and utilized to stimulate recipient HaCaT cells at a concentration of 20 μg/mL for an additional 24 h.

### 2.3 Isolation of exosomes

Exosomes were isolated from HaCaT cells, as well as peripheral blood mononuclear cells (PBMCs) obtained from both healthy controls and patients with psoriasis. The Exo-spin™ mini exosome purification kit (Cell Guidance Systems, St. Louis, MO, United States) was employed following the manufacturer’s protocol. Starting sample (50 mL) underwent centrifugation at 300 × g for 10 min to remove cells. The resulting supernatant was then transferred to a new microcentrifuge tube and centrifuged at 16,000 × *g* for 30 min. Subsequently, the supernatant was transferred to another microcentrifuge tube, and Exo-spin™ Buffer was added at a 2:1 ratio. After gently mixing by inversion, the tube was incubated at 4°C overnight. The mixture was then centrifuged at 16,000 g for 1 h, and the resulting pellet containing exosomes was resuspended in 100 µL of PBS. This resuspended exosome-containing pellet (100 µL) was applied to the top of the Exo-spin™ column, placed into a 1.5 mL microcentrifuge tube, and allowed to enter the column matrix under gravity. Subsequently, 180 µL of PBS was added to the top of the column and eluted. The Exo-spin™ column was then removed from the sample collection tube, and the sample collection tubes containing isolated exosomes were centrifuged at 100 × g for 30 s.

### 2.4 MTT assay

Cytotoxicity was assessed using a 3-(4,5-dimethylthiazol-2-yl)-2,5-diphenyltetrazolium bromide (MTT) assay (Sigma-Aldrich). Psoriatic PBMCs at a concentration of 1.5 × 10^6^ μg/mL were seeded into 96-well plates, incubated overnight, and starved for 24 h. Subsequently, varying concentrations (0, 0.1, 10, 20, 50, and 100 nM) of BaP were added, and the cells were cultured in serum-free DMEM for an additional 24 h. Following incubation, cells were washed with phosphate-buffered saline (PBS), treated with MTT solution for 4 h at 37°C, and the formazan crystals were dissolved in 500 µL of dimethyl sulfoxide (DMSO). Absorbance at 570 nm was measured using a microplate reader (Molecular Devices, Sunnyvale, CA, United States).

### 2.5 Nanoparticle tracking analysis (NTA)

The particle size distribution and exosome count in the conditioned medium were determined using NanoSight (NS300, Malvern, United Kingdom) equipped with a 488 nm laser. The sample was loaded into the chamber using a 1 mL disposable syringe. A 40-s video recorded all events for subsequent analysis using NTA software, based on Brownian motion. Exosomes were diluted in PBS until an optimal number was detected, and measurements were performed four times for each sample. The data represent both the number of exosomes and their size distribution.

### 2.6 Exosome uptake assay

Exosomes were labeled with the green fluorescent dye Vybrant DiO cell-labeling solution (Sigma-Aldrich). After washing with PBS, cells were fixed with 4% paraformaldehyde for 15 min. Subsequently, cells were permeabilized with 0.2% Triton X-100 in 1% bovine serum albumin (BSA) for 10 min and blocked with 3% BSA for 1 hour at room temperature. Vybrant DiO cell-labeling solution (Sigma-Aldrich) was added to the experimental group and incubated at 37°C for 2 h. Exosome uptake was visualized using fluorescence microscopy with the Leica Microsystems DFi8 LASX software light microscope (Leica, Wetzlar, Germany).

### 2.7 Quantitative PCR

Total RNA was extracted using the RNeasy Plus Mini Kit (Qiagen, Hilden, Germany). Subsequently, the First Strand cDNA synthesis kit (Roche Diagnostics, Mannheim, Germany) was employed to synthesize cDNA from 1 µg of total RNA. Quantitative reverse transcriptase-PCR was conducted in triplicate using TaqMan Master Mix (Applied Biosystems) and a Real-Time PCR System (Applied Biosystems). The following primers were used for mRNA detection in this study: AhR (TaqMan Assay ID Hs 00169233_m1), CYP1A1 (TaqMan Assay ID Hs 1054794_m1), CXCL1 (TaqMan Assay ID Hs 00236937_m1), CXCL5 (TaqMan Assay ID Hs 00607029_m1), TNF-α (TaqMan Assay ID Hs 00174128_m1), IL-1β (TaqMan Assay ID Hs 1555410_m1), IL-6 (TaqMan Assay ID Hs 00174131_m1), IL-8 (TaqMan Assay ID Bt 3211906_m1), and GAPDH (TaqMan Assay ID 02758991_m1). Normalization of mRNA levels for AhR, CYP1A1, CXCL1, CXCL5, TNF-α, IL-1β, IL-6, and IL-8 was performed against glyceraldehyde-3-phosphate dehydrogenase (GAPDH). Fold changes were calculated using the ΔΔCt method, and relative quantification was conducted using a LightCycler 96 instrument (Roche Diagnostics).

### 2.8 Western blotting

Cells were harvested in Pro-Prep lysis buffer (Intron, Seoul, Korea), supplemented with a protease inhibitor cocktail (Roche Diagnostics). Protein concentrations were determined using a copper (II) sulfate solution in bicinchoninic acid (Sigma‒Aldrich). Equal amounts of protein (20 µg) were separated by 10% SDS‒PAGE, transferred to enhanced chemiluminescence (ECL) nitrocellulose membranes (GE Healthcare, Buckinghamshire, United Kingdom), and blocked for 1 h with 5% skim milk in TBST. Membranes were incubated overnight at 4°C with specific primary antibodies, including rabbit anti-CD63 (1:1000, Abcam, Cambridge, United Kingdom), rabbit anti-CD9 (1:1000, Abcam), rabbit anti-HSP70 (1:1000, Abcam), rabbit anti-Alix (1:1000, Abcam), rabbit anti-TSG101 (1:1000, Abcam), rabbit anti-AhR (1:1000, Abcam), rabbit anti-CYP1A1 (1:1000, Abcam), rabbit anti-CXCL1 (1:1000, Abcam), rabbit anti-CXCL5 (1:1000, Abcam), rabbit anti-TNF-α (1:1000, Abcam), rabbit anti-IL-1β (1:1000, Abcam), rabbit anti-AhR (1:1000, Abcam), rabbit anti-IL-6 (1:1000, Abcam), rabbit anti-IL-8 (1:1000, Abcam), rabbit anti-ERK (1:1000, Abcam), rabbit anti-phospho ERK (1:1000, Abcam), rabbit anti-P65 (1:1000, Abcam), rabbit anti-phospho P65 (1:1000, Abcam), rabbit anti-P38 (1:1000, Abcam), and rabbit anti-phospho P38 (1:1000, Abcam) antibodies. Primary antibodies were detected using horseradish peroxidase-conjugated secondary antibodies (goat anti-rabbit, 1:1000; Abcam) and chemiluminescent luminol (LUMINOGRAPH II; Atto, Tokyo, Japan). Immunocomplexes were visualized using an enhanced horseradish peroxidase/luminol chemiluminescence system (ECL Plus; Amersham International PLC, Little Chalfont, United Kingdom). GAPDH served as the loading control for Western blot analysis.

### 2.9 Transfection of siRNAs (small interfering RNAs) specific for AhR

siRNAs targeting AhR (AhR siRNA; Ambion; Thermo Fisher Scientific, Waltham, MA, United States) and a scrambled sequence control siRNA, which would not induce specific degradation of any cellular mRNA, were obtained from Ambion (Ambion; Thermo Fisher Scientific). HaCaT cells, cultured in 6-well plates, were incubated for 24 h in 0.5 mL of culture medium with a mixture containing 5 nM siRNA and 3 µL of Lipofectamine RNAiMAX (Invitrogen; Thermo Fisher Scientific). Transfection was carried out after 24 h of culture using siRNA duplexes targeting the AhR mRNA sequence or control siRNA.

### 2.10 Flow cytometry

For surface staining, isolated PBMCs were stained with an APC anti-human CD4 antibody (R&D Systems, Minneapolis, MN, United States). For intracellular staining, cells were fixed with Cytofix buffer, permeabilized with Cytoperm solution (eBioscience, San Diego, CA, United States), and stained with Percp-cy5.5**-**anti-human IL-17A and FITC-anti-human IFN-γ (R&D Systems). Labeled cells were quantified using a Cyto FLEX Flow Cytometer (Beckman Coulter, CA, United States), and the data were analyzed using Cytex pert 2.4 (Beckman). The gating strategy was as follows: cells were first gated for lymphocytes in a forward/side scatter plot (FCS-A and SSC-A), and then gated for the separation of CD4^+^ T cells. CD4^+^ T cells were further gated for the identification of IFN-γ-producing CD4^+^ T cells (Th1 phenotype) and IL-17A-producing CD4^+^ T cells (Th17 phenotype).

### 2.11 Cell immunofluorescence

Human peripheral PBMCs were seeded on coverslips and allowed to adhere overnight. After washing with PBS, cells were fixed with 4% paraformaldehyde for 15 min, permeabilized with 0.2% Triton X-100 in 1% bovine serum albumin (BSA) for 10 min, blocked with 3% BSA for 1 hour at room temperature, and incubated overnight at 4°C with anti-IL6 (Abcam, Cambridge, United Kingdom), anti-IL17A (Abcam), anti-IL22 (Abcam), anti-IL23 (Abcam), and anti-IFN-γ (Abcam). Subsequently, Cells were immunolabeled using a combination of fluorescein-5-isothiocyanate (FITC)-conjugated goat anti-rabbit IgG (Abcam) at 1:200 for 1 h. The heavy chain of FITC-conjugated goat anti-rabbit IgG served as the marker for immunofluorescence staining. For nuclear counterstaining, mounting medium was used along with DAPI (Vector Laboratories, Burlingame, CA, United States). Fluorescent images were captured using a Leica Microsystems DFi8 with LASX software light microscopy (Leica, Wetzlar, Germany). The fluorescence intensity was semi-quantitatively analyzed using LAS X software (Leica, Wetzlar, Germany). The results are presented as the mean optical density with standard deviation based on three different digital images.

### 2.12 Statistical analyses

Statistical analyses were performed using GraphPad Prism version 5.01 (GraphPad Software, San Diego, CA, United States). Data were analyzed using the Student’s t-test and one-way analysis of variance with Tukey’s *post hoc* test. Statistical significance was set at *p* < 0.05 significant.

## 3 Result

### 3.1 Characterization of exosomes and exosome uptake analysis

**FIGURE 1:**
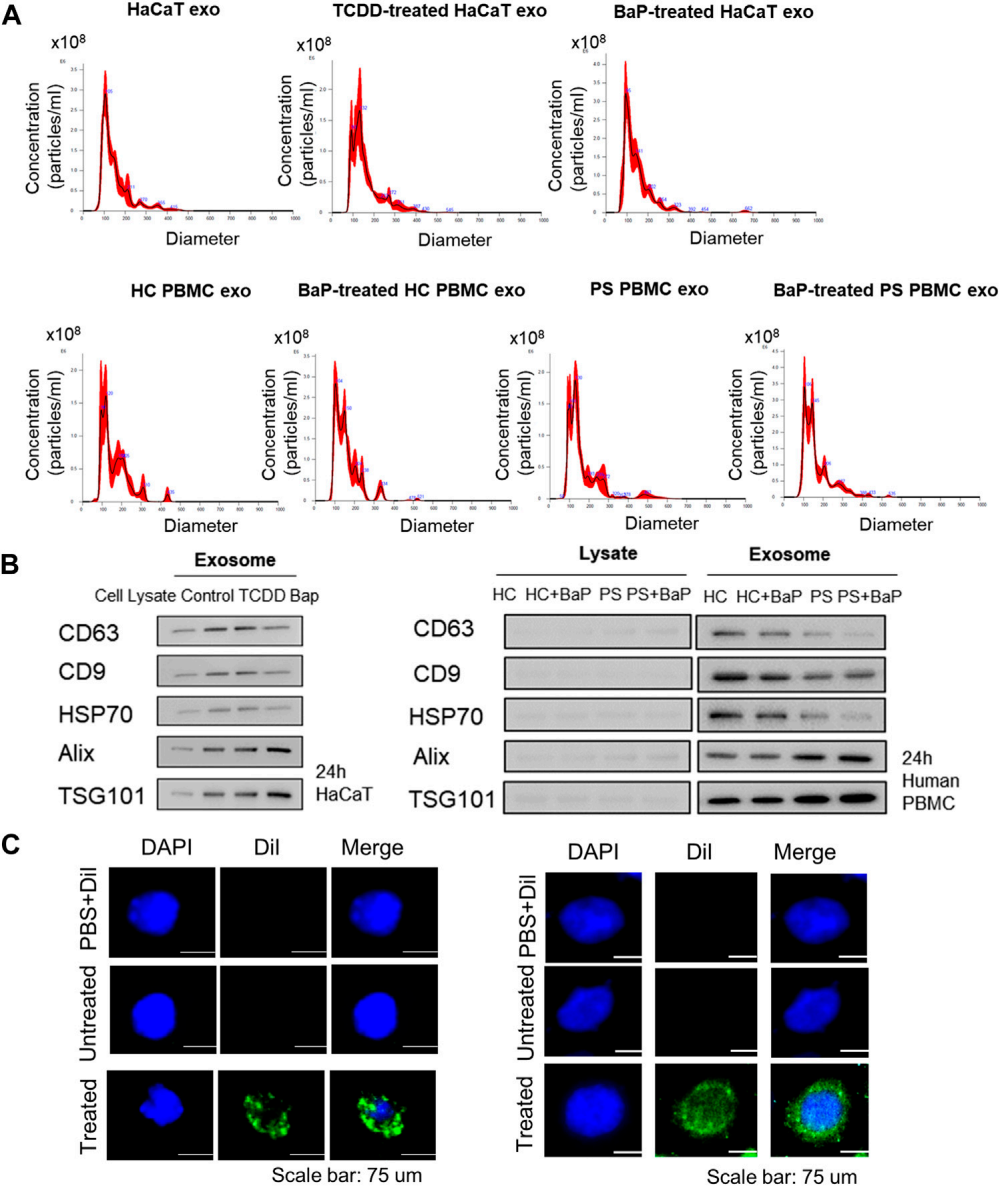
Characterization of exosomes derived from HaCaT cells and PBMCs and exosome uptake analysis. **(A)** Size distribution of exosomes by nanoparticle tracking analysis (*n* = 4). **(B)** The expression of CD63, CD9, HSP70, Alix and TSG101 in exosomes and the corresponding total cell lysates. The relative expression was normalized to GAPDH (*n* = 3). The densitometric value of each independent band was compared with the total cell lysate control. **(C)** The representative immunofluorescence images of exosomes derived from BaP-treated HaCaT cells-treated recipient HaCaT cells or BaP-treated PS PBMC-treated recipient HC PBMCs. Nuclei were counterstained with DAPI (blue). Scale bar, 75 μm. exo, exosomes, BaP, benzo [a]pyrene. PS, psoriasis patients, HC, healthy controls, PBMC, peripheral blood mononuclear cell.

Exosomes were extracted from untreated HaCaT cells, TCDD-treated HaCaT cells, BaP-treated HaCaT cells, HC PBMCs, BaP-treated HC PBMCs, PBMCs from patients with psoriasis (PS), and BaP-treated PBMCs from PS. Nanoparticle tracking analysis verified average diameters of 145.9 ± 65.1 nm, 149.9 ± 59.5 nm, 142.3 ± 56.4 nm, 153 nm ± 49, 153.8 ± 58.1 nm, 156.1 ± 59 nm and 160.4 ± 67.4 nm across all groups (*n* = 4 each) (Figure 1A). All exosome groups exhibited expression of key exosomal markers (CD63, CD9, HSP70, Alix, and TSG101) when compared to total cell lysates. Notably, the exosomes from BaP-treated HaCaT cells showed elevated levels of Tsg101 and Alix, alongside reduced CD63 levels compared to non-treated cells. Exosomes isolated from PS PBMCs displayed lower CD63, CD9, and HSP70 levels but higher Alix and TSG101 expression compared to healthy controls (Figure 1B, Supplementary Figure S3). To validate exosome uptake, we labeled exosomes from BaP-treated HaCaT cells or BaP-treated PS PBMCs with DiI and introduced them to recipient HaCaT cells or HC PBMCs for 2 h. Efficient exosome uptake was observed in both HaCaT cells and HC PBMCs (Figure 1C).

### 3.2 Induction of proinflammatory cytokines and chemokines in HaCaT cells by exosomes from BaP- or TCDD-treated cells

**FIGURE 2:**
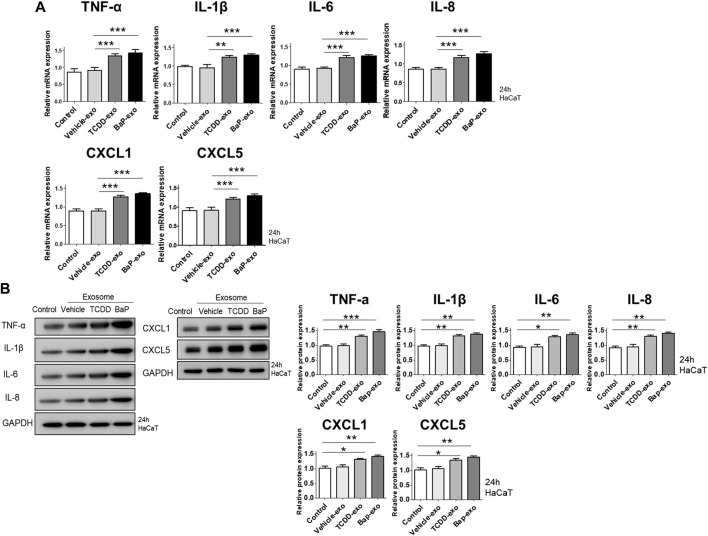
The effects of exosomes derived from BaP- or TCDD-treated HaCaT cells on proinflammatory cytokine/chemokine mRNA or protein expression in recipient HaCaT cells. **(A)** The mRNA expression of TNF-α, IL-1β, IL-6, IL-8, CXCL1 and CXCL5. Statistical analysis was performed by one-way ANOVA followed by Tukey’s multiple comparison test. Data represent the mean ± S.D. of 3 independent experiments. **p < 0.01 and ***p < 0.001. **(B)** Immunoblotting of TNF-α, IL-1β, IL-6, IL-8, CXCL1 and CXCL5. The relative expression was normalized to GAPDH (*n* = 3). The relative intensities of western blots were compared with the control and measured by ImageJ software. Statistical analysis was performed by one-way ANOVA followed by Tukey’s multiple comparison test. Data represent the mean ± S.D. of 3 independent experiments. *p < 0.05, **p < 0.01 and ***p < 0.001.

**FIGURE 3:**
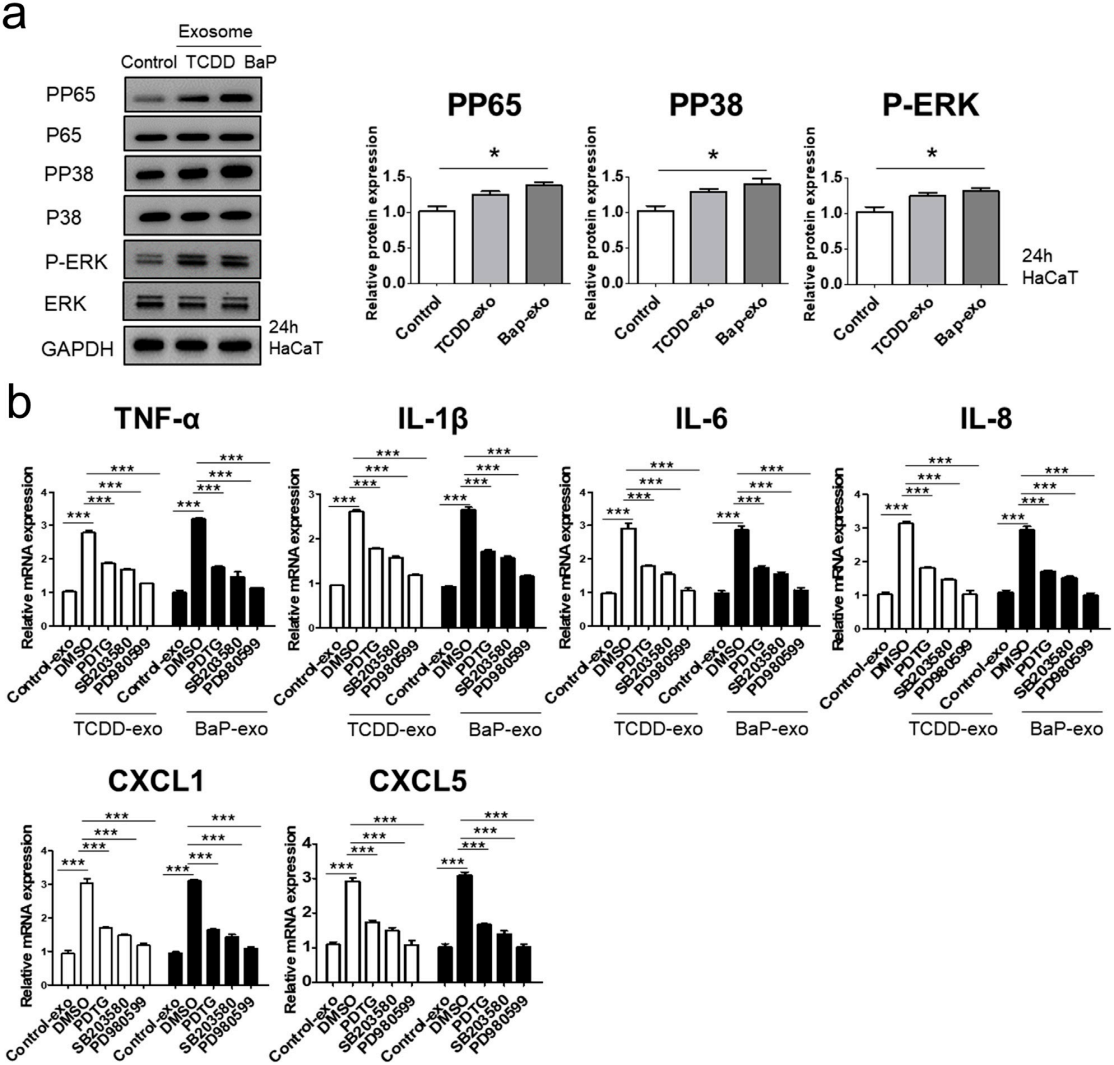
The effects of exosomes derived from BaP- or TCDD-treated HaCaT cells on the p65/NF-κB, p38/MAPK and ERK/MAPK signaling pathways in recipient HaCaT cells. **(A)** P-P65, P-P38 and P-ERK expression treated with exosomes derived from BaP- or TCDD-treated HaCaT cells by Western blotting. The relative expression was normalized to GAPDH (*n* = 3). The relative intensity of each band was quantified by densitometric scan. **(B)** TNF-α, IL-1β, IL-6, IL-8, CXCL1, and CXCL5 expression with the exosome-derived from BaP- or TCDD-treated HaCaT cell in the absence or presence of inhibitors of P65 (PDTC, 10 µM), P38 (SB203580, 10 µM), or ERK (PD980599, 5 µM) by quantitative PCR. Statistics: mean ± S.D. (*n* = 3). Statistical significance was determined by one-way ANOVA followed by Tukey’s multiple comparison test. **p < 0.01 and ***p < 0.001. PDTC, pyrrolidine dithiocarbamate, exo, exosome, BaP, benzo [a]pyrene.

We evaluated the ability of exosomes from BaP- or TCDD-treated HaCaT cells to trigger proinflammatory cytokine and chemokine production in recipient HaCaT cells. We found that exosomes from BaP- or TCDD-treated HaCaT cells led to higher mRNA and protein expression of TNF-α, IL-1β, IL-6, CXCL1, and CXCL5 compared to untreated cells (Figures 2A, B, Supplementary Figure S3). Overall, these exosomes induced proinflammatory cytokine and chemokine production in recipient HaCaT cells. To further elucidate the signaling pathways involved in the proinflammatory response induced by TCDD- or BaP-treated HaCaT cell-derived exosomes, we assessed the phosphorylation of P65/NF-κB, P38/MAPK, and extracellular signal-regulated kinase (ERK)/MAPK in recipient HaCaT cells. Treatment with exosomes from TCDD- or BaP-treated HaCaT cells increased phosphorylation levels of these signaling pathways compared to the control (Figure 3A, Supplementary Figure S3). To confirm the involvement of P65/NF-κB, P38/MAPK, and ERK/MAPK signaling in the observed changes, we pretreated HaCaT cells with inhibitors for 1 h before stimulating the cells with exosomes from TCDD (10 nM) or BaP (0.5 µM)-treated HaCaT cells (20 μg/mL) for 24 h. Inhibition of P65, P38, and ERK significantly reduced the upregulation of TNF-α, IL-1β, IL-6, IL-8, CXCL1, and CXCL5 expression by exosomes from BaP- or TCDD-treated HaCaT cells in recipient HaCaT cells, confirming the involvement of the P65/NF-κB and MAPK targeting P38/ERK signaling pathways (Figure 3B).

### 3.3 Upregulation of cytokine and chemokine expression in recipient HaCaT cells induced by BaP- or TCDD-stimulated HaCaT cell-derived exosomes via the AhR signaling pathway

**FIGURE 4:**
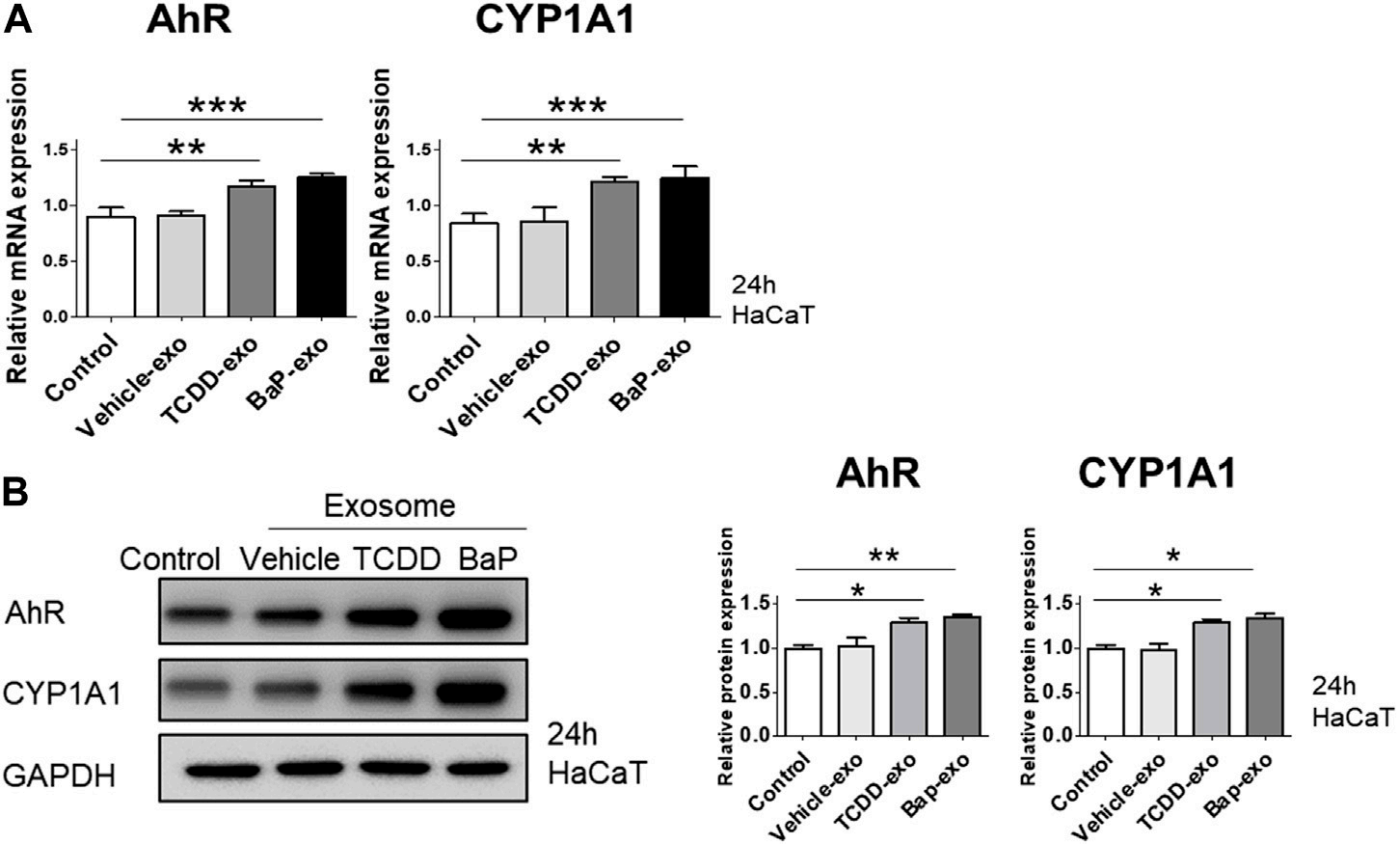
The effects of exosomes derived from BaP- or TCDD-treated HaCaT cells on the AhR and CYP1A1 expression in recipient HaCaT cells. **(A)** The treatment of recipient HaCaT cells with exosomes derived from BaP- or TCDD-treated HaCaT cells increased AhR and CYP1A1 mRNA expression. Statistical analysis was performed by one-way ANOVA followed by Tukey’s multiple comparison test. Data represent the mean ± SD of 3 independent experiments. **p < 0.01 and ***p < 0.001. **(B)** The protein expression of AhR and CYP1A1. The relative expression was normalized to that of GAPDH (*n* = 3). The relative intensities were compared with the control.

To assess the capability of BaP- or TCDD-treated HaCaT cell-derived exosomes in activating AhR in recipient HaCaT cells, we exposed HaCaT cells to these exosomes for 24 h and subsequently measured the expression of AhR and CYP1A1. In comparison to the control group, both TCDD-treated and BaP-treated HaCaT cell-derived exosome groups exhibited increased mRNA and protein expression of AhR and CYP1A1 (Figures 4A, B, Supplementary Figure S3). Thus, we observed that TCDD- or BaP-treated HaCaT cell-derived exosomes induced AhR activation in recipient HaCaT cells.

**FIGURE 5:**
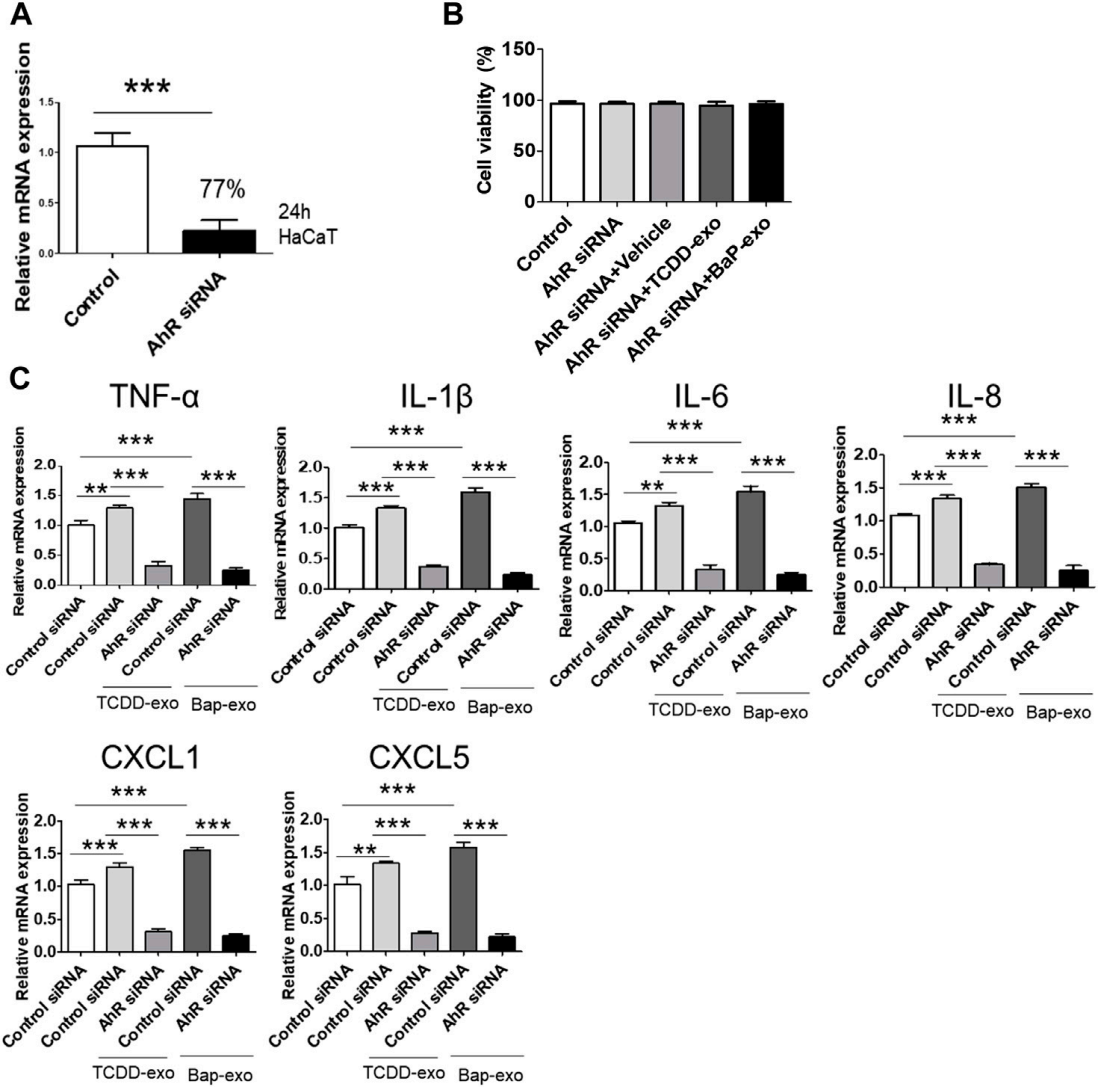
The Upregulation of Cytokine and Chemokine Expression in Recipient HaCaT Cells Treated with BaP- or TCDD-Stimulated HaCaT Cell-Derived Exosomes was dependent on AhR signaling pathway. **(A)** A 77% reduction in AhR mRNA was shown in the AhR siRNA-transfected cells compared to the nontransfected cells. HaCaT cells were transfected with AhR siRNA for 24 h. **(B)** Cell viability of AhR siRNA transfected HaCaT cells by MTT assay. Statistical analysis was performed by one-way ANOVA followed by Tukey’s multiple comparison test. Data represent the mean ± SD of 3 independent experiments. **(C)** AhR silencing attenuated the upregulation induced by exosomes from TCDD-treated HaCaT cells or BaP-treated HaCaT cells on TNF-α, IL-1β, IL-6, IL-8, CXCL1, and CXCL5 in recipient HaCaT cells. Data represent the mean ± S.D. (*n* = 3). Statistical analysis was performed by one-way ANOVA followed by Tukey’s multiple comparison test. **p < 0.01 and ***p < 0.001. exo, exosome, BaP, benzo [a]pyrene.

To further corroborate the influence of AhR on proinflammatory cytokine and chemokine production in recipient HaCaT cells treated with BaP- or TCDD-stimulated HaCaT cell-derived exosomes, we conducted AhR blockade experiments using siRNA oligonucleotides (Figure 5A). HaCaT cells were transfected with AhR siRNA for 24 h and subsequently stimulated with TCDD (10 nM)-treated HaCaT cell-derived exosomes (20 μg/μL) or BaP (0.5 µM)-treated HaCaT cell-derived exosomes (20 μg/μL) for an additional 24 h. To ensure the viability of transfected cells, we performed the MTT assay, which indicated that AhR siRNA transfection did not affect cell viability (Figure 5B). The silencing of AhR attenuated the upregulation of proinflammatory cytokines and chemokines induced by exosomes from TCDD-treated or BaP-treated HaCaT cells in recipient HaCaT cells, suggesting a role for AhR in mediating these responses (Figure 5C).

### 3.4 Treatment with BaP-Stimulated PS PBMC-derived exosomes induced psoriatic cytokine expression via AhR activation in recipient HC PBMCs

**FIGURE 6:**
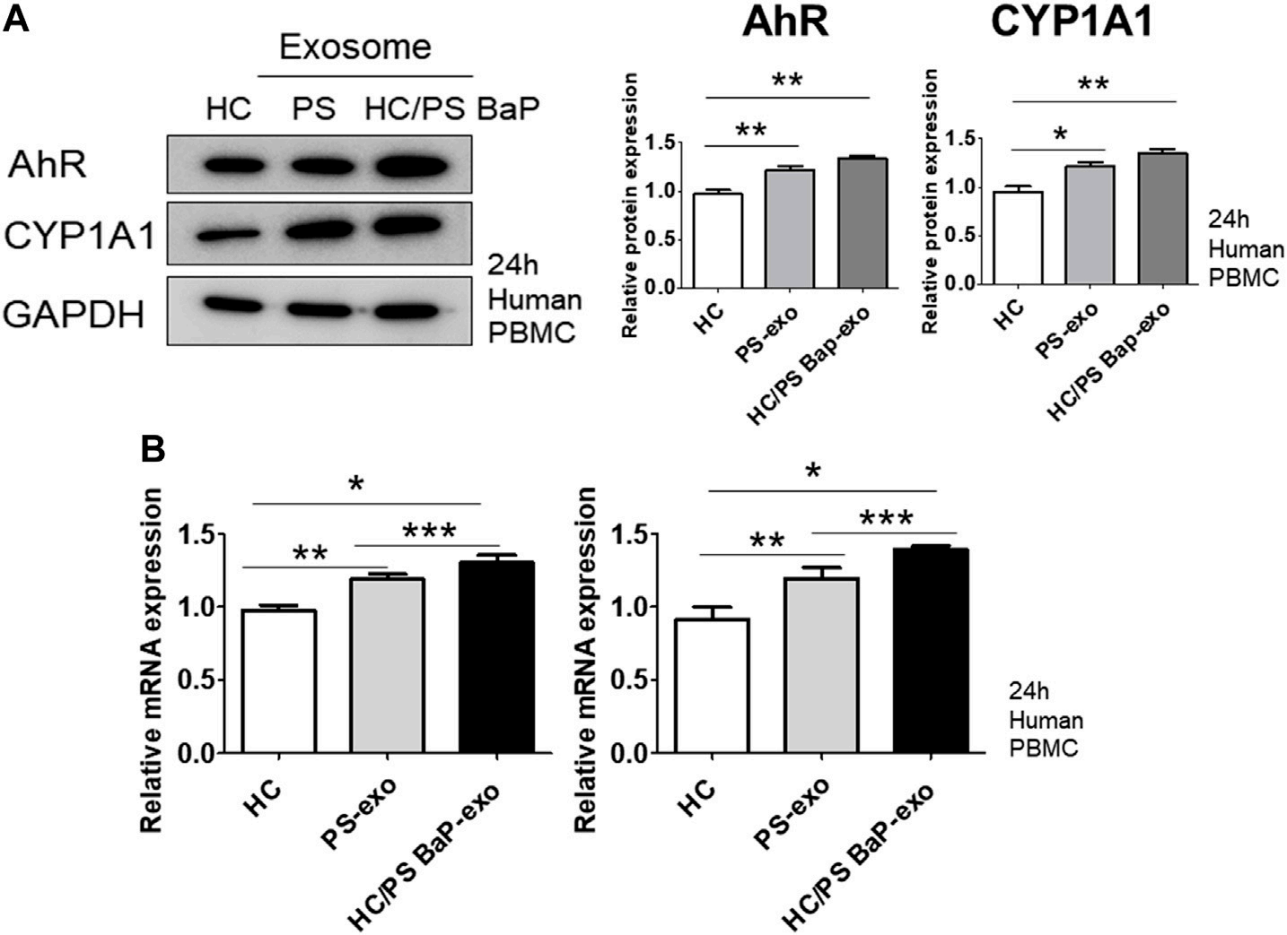
The effects of exosomes derived from BaP-treated PS PBMCs on AhR and CYP1A1 expression in recipient HC PBMCs. **(A)** Representative images of western blots for AhR and CYP1A1. The relative expression was normalized to GAPDH. The results are representative of three independent experiments. The relative intensities of western blots were measured by ImageJ. **(B)** mRNA expression of AhR and CYP1A1. Statistical significance was determined by one-way ANOVA followed by Tukey’s multiple comparison test. Data represent the mean ± S.D. of 3 independent experiments. *p < 0.05, **p < 0.01 and ***p < 0.001.

**FIGURE 7:**
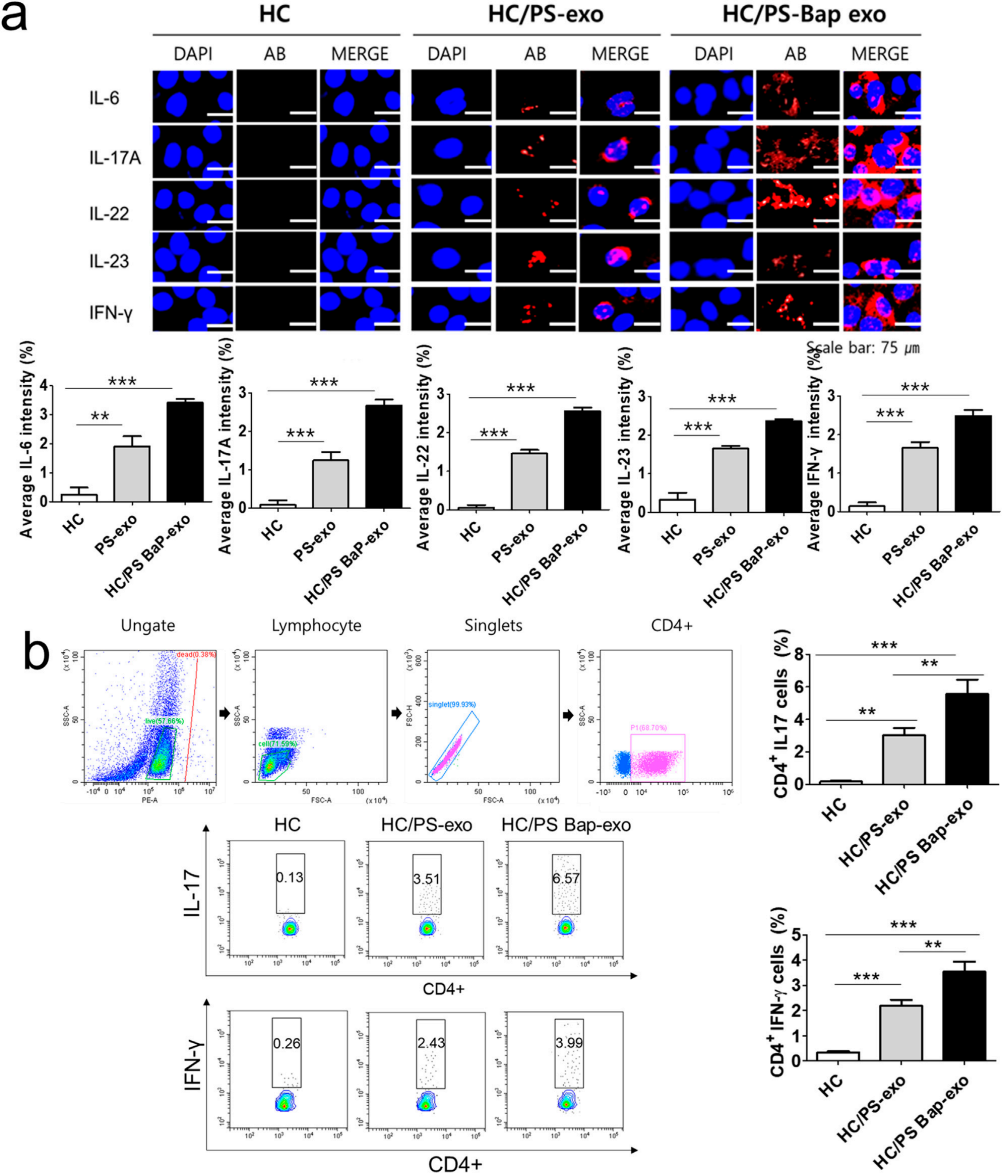
The effects of exosomes derived from BaP-treated PS PBMCs on proinflammatory cytokine expression, and the distribution of IL-17A-positive and IFN-γ-positive CD4^+^ T cells in recipient HC PBMCs. **(A)** The expressions of IL-6, IL-17A, IL-22, IL-23, and IFN-γ by immunofluorescence. Results are a representation of one sample of each group (PS = 3, HC = 3). Scale bar = 75 µm. The fluorescence intensity was semi-quantitatively analyzed and the results are presented as the mean optical density with standard deviation based on three different digital images. Statistical significance was determined by one-way ANOVA followed by Tukey’s multiple comparison test. ***p < 0.001 **(B)** Representative dot plots and the percentages of IL-17A-positive and IFN-γ-positive CD4^+^ T cells. The percentage of IL-17A-positive and IFN-γ-positive CD4^+^ T cells by flow cytometric analysis. The percentage of dead cells in IL-17 and CD4 was 1.86% for HC, 1.09% for HC/PS-exo, and 0.21% for HC/PS Bap-exo. For INF-y and CD4, the percentages were 0.38% for both HC and HC/PS-exo, and 0.11% for HC/PS Bap-exo and 0.57%. Results are a representation of one sample of each group (PS = 3, HC = 3). Statistics: mean ± S.D. **p < 0.05 and ***p < 0.001. exo, exosome, BaP, benzo [a]pyrene, PS, psoriasis patient, HC, healthy control.

Next, we investigated the impact of 10 µM BaP-treated PS PBMC-derived exosomes (20 μg/mL) on recipient HC PBMCs over a 24-h period. The assessment included the measurement of AhR, CYP1A1 expression, proinflammatory cytokines, and the distribution of CD4+IL-17 cells and CD4+IFN-γ cells. The concentration of 10 µM BaP in PBMC experiments was determined based on the results of the MTT assay (Supplementary Figure S1). HC PBMCs treated with PS PBMC-derived exosomes (20 μg/mL) exhibited elevated protein and mRNA expression levels of AhR and CYP1A1 compared to untreated HC PBMCs. Furthermore, BaP-treated PS PBMC-derived exosomes increased the protein and mRNA expression of AhR and CYP1A1 in recipient HC PBMCs compared to HC PBMCs treated with PS PBMC-derived exosomes alone (Figures 6A, B, Supplementary Figure S3). In the immunofluorescence analysis, BaP-treated PS PBMC-derived exosomes heightened the staining intensities of IL-6, IL-17A, IL-22, IL-23, and IFN-γ compared to untreated HC PBMCs or HC PBMCs treated with PS PBMC-derived exosomes. As anticipated, HC PBMCs treated with PS PBMC-derived exosomes showed higher levels of all cytokines than untreated HC PBMCs (Figure 7A). About 50 percentages of lymphocytes was presented in relation to total PBMCs. We observed that exosomes derived from BaP-treated PS PBMCs enhanced the staining intensities of IL-6, IL-17A, IL-22, IL-23, and IFN-γ in CD4^+^ lymphocytes compared to the untreated HC CD4^+^ lymphocytes group or HC CD4^+^ lymphocytes treated with PS PBMC-derived exosomes (Supplementary Figure S2). The distribution of CD4+IL-17 cells and CD4+IFN-γ cells significantly increased in recipient HC PBMCs treated with BaP-treated PS PBMC-derived exosomes compared to HC PBMCs or HC PBMCs treated with PS PBMC-derived exosomes alone. Additionally, HC PBMCs treated with PS PBMC-derived exosomes exhibited a higher distribution of CD4+IL-17 cells and CD4+IFN-γ cells compared to HS PBMCs (Figure 7B). In summary, these findings demonstrate that BaP-treated PS PBMC-derived exosomes induce the expression of TH1 and TH17 cytokines and promote the differentiation of TH1 and TH17 cells in recipient PBMCs.

## 4 Discussion

This study examines the impact of BaP and TCDD on exosome function and their role in immune-related changes in recipient keratinocytes and PBMCs. Treating HaCaT cells with BaP or TCDD induces exosomes from the treated keratinocytes to upregulate proinflammatory cytokines and chemokines in recipient keratinocytes through AhR activation. The upregulation of these inflammatory molecules involves the p65NF-κB/p38MAPK/ERK signaling pathway. Moreover, exosomes from BaP-stimulated PBMCs of psoriatic patients increase the expression of proinflammatory cytokines (IL-6, IL-17A, IL-22, IL-23, and IFN-γ) and promote Th1/Th17 cell distribution in recipient PBMCs via AhR activation. This reveals that environmental toxicants BaP and TCDD induce psoriatic inflammatory reactions in human keratinocytes and PBMCs through exosome-mediated cell-to-cell communication.

Psoriasis, a common immune-mediated inflammatory cutaneous disorder, is associated with systemic diseases encompassing arthritic, metabolic, cardiovascular, and psychological comorbidities (Griffiths et al., 2021). Its complex pathogenesis involves abnormal activation of the innate and adaptive immune systems, with the IL-23/IL-17 axis playing a central role. This axis leads to excessive keratinocyte proliferation (Lowes et al., 2008). The interplay between genetic susceptibility and environmental stimuli evokes the development or aggravation of psoriasis (Griffiths et al., 2021). While the role of genetic factors in psoriasis pathogenesis has been well elucidated (Tang et al., 2014; Tsoi et al., 2017), the detailed molecular mechanisms underlying the influence of environmental factors on psoriasis remain unknown. Cigarette smoking, a well-known environmental factor for psoriasis (Fortes et al., 2005; Jankovic et al., 2009), is intriguingly linked to BaP, a major PAH in smoke fume. BaP elicits proinflammatory psoriatic molecules via the p65NF-κB/p38MAPK/ERK pathways by transferring exosomes from parent keratinocytes to recipient keratinocytes. Additionally, BaP affects exosomes from peripheral PBMCs of psoriasis patients, triggering exacerbated Th1/Th17 cytokine production and Th1/Th17 CD4^+^ T-cell differentiation in recipient peripheral PBMCs from normal controls.

AhR plays critical roles in inflammatory and immune-mediated cutaneous diseases, including chloracne, atopic dermatitis, and psoriasis (Rothhammer and Quintana, 2019; Furue, 2020). Activated by various ligands, including endogenous L-tryptophan-derived ligands, exogenous dietary ligands, microbial-derived ligands, and environmental xenobiotic agents (e.g., TCDD and BaP), AhR is implicated in detrimental effects on human organs, including the skin. Growing evidence suggests that exposure to TCDD or BaP induces pathological responses in the skin, leading to chronic inflammatory skin diseases such as psoriasis through AhR activation (Tsuji et al., 2011; Kim et al., 2014; Smith et al., 2017a). TCDD induces an increase in CXCL5 expression via AhR activation in primary mouse keratinocytes (Smith et al., 2017a), while *in vitro* studies show that TCDD elevates the secretion of IL-6 and IL-8 in normal human epidermal keratinocytes (Kim et al., 2014). BaP triggers an increase in IL-8 production in an AhR-reactive oxygen species (ROS)-dependent manner in human keratinocytes (Tsuji et al., 2011). Our study found that exosomes isolated from TCDD- or BaP-treated keratinocytes exhibit proinflammatory reactions through AhR activation in recipient cells. AhR silencing attenuates the upregulation of proinflammatory cytokines and chemokines in recipient keratinocytes induced by exosomes from BaP- or TCDD-treated keratinocytes, suggesting AhR’s involvement in the expression of psoriatic inflammatory markers, including TNF-α, IL-1β, IL-6, IL-8, CXCL1, and CXCL5, by BaP or TCDD.

AhR plays a pivotal role as a modulator in the context of inflammatory and autoimmune Th17 cell populations, responding to specific ligands or the cellular microenvironment. Interestingly, diverse ligands prompt AhR to engage distinct transcriptional partners, leading to AhR binding to varied DNA target sequences and resulting in distinct biological reactions (Quintana et al., 2008; Duarte et al., 2013). Activation of AhR by TCDD has been shown to enhance Th17 cell differentiation *in vitro* (Duarte et al., 2013). Moreover, Kim et al. observed that TCDD exacerbates skin lesions in a psoriasis-like mouse model by inducing the production of Th17 cytokines, such as IL-17A and IL-22 (Kim et al., 2021).

In our study, we found that BaP-treated PBMCs from psoriasis patient-derived exosomes promote Th17/Th1 cell differentiation in recipient PBMCs from healthy controls. This effect was compared to the response observed in healthy control PBMCs stimulated by non-treated PBMCs from psoriasis patient-derived exosomes. Furthermore, BaP-treated PBMCs from psoriasis patient-derived exosomes upregulated the expression of proinflammatory cytokines, including IL-6, IL-17A, IL-22, IL-23, and IFN-γ, in recipient PBMCs from healthy controls, in contrast to HC PBMCs treated with PBMCs from non-treated psoriasis patient-derived exosomes. While previous studies, including our current investigation, have demonstrated that environmental toxicants like TCDD or BaP induce proinflammatory reactions, it is noteworthy that endogenous AhR ligands, such as FICZ, and the direct binding of tapinarof to AhR yield anti-inflammatory responses in psoriasis-like models (Di Meglio et al., 2014).

Furthermore, tapinarof exhibits direct binding to the aryl hydrocarbon receptor (AhR), leading to a reduction in the production of inflammatory cytokines, including IL-17A, IL-17F, IL-22, IL-23, and IL-1β, as demonstrated in a psoriasis-like mouse model (Smith et al., 2017b). Clinical trials have substantiated the efficacy of topical tapinarof in patients with psoriasis and atopic dermatitis (Lebwohl et al., 2021; Strober et al., 2022). The distinct biological functions of AhR, mediated by various ligands, are hypothesized to be influenced by divergent AhR activation pathways governed by regulatory mechanisms (Fernandez-Gallego et al., 2021). Controlled AhR activation through physiological ligands, such as FICZ, supports the preservation of skin homeostasis and anti-inflammatory reactions. Conversely, prolonged and heightened uncontrolled AhR activation induced by pathological ligands, including TCDD and BaP, elicits inflammatory responses associated with cutaneous inflammatory diseases, such as psoriasis.

Exosomes, derived from both non-immune and immune cells, play a pivotal role in regulating immune reactions associated with inflammatory diseases (Xiong et al., 2021). Despite suggestions regarding the involvement of exosomes in the pathogenesis of psoriasis, limited studies have been conducted. Cheung et al. demonstrated that exosomes can serve as crucial carriers of non-peptide antigens, such as neolipids, acting as significant regulators of T cells (Cheung et al., 2016). In their study, Cheung et al. showed that exosomes containing Phospholipase A2 (PLA2) from LAD2 mast cells induced the production of IL-17A and IL-22 by CD1a-autoreactive T cells from psoriasis patients (Cheung et al., 2016). Notably, our study found that BaP stimulation in exosomes from PBMCs of patients with psoriasis triggered inflammatory reactions involving changes in psoriatic cytokine expression and Th1/Th17 CD4^+^ T-cell distribution in recipient PBMCs from healthy controls. These data imply that BaP may amplify these proinflammatory responses in immune cells through exosome transfer. In addition to immune cells, keratinocytes play key roles in the development of psoriasis, characterized by abnormalities in keratinocyte proliferation and differentiation (Ni and Lai, 2020). Our study further demonstrated that BaP-treated keratinocyte exosomes induced psoriatic inflammatory reactions in recipient keratinocytes. Exosomes derived from BaP-stimulated human keratinocytes triggered the expression of psoriatic cytokines and chemokines, including TNF-α, IL-1β, IL-6, IL-8, CXCL1, and CXCL5, through the activation of the NF-κB and MAPK pathways in recipient human keratinocytes. Notably, these inflammatory responses were mediated by the AhR signaling pathway. Two prior studies have highlighted the role of exosomes as cell-to-cell messengers between keratinocytes and immune cells in psoriasis pathogenesis (Jiang et al., 2019; Shao et al., 2019). Jiang et al. reported that psoriasis-like cytokine-treated keratinocyte exosomes increased the expression of TNF-α, IL-6, and IL-8 in neutrophils through NF-κB/MAPK signaling (Jiang et al., 2019). Similarly, Shao et al. identified that exosomes from neutrophils of generalized pustular psoriasis patients increased the expression of inflammatory molecules, such as IL-1β, IL-36, IL-18, and TNF-α, in recipient keratinocytes (Shao et al., 2019). Collectively, based on previous studies and our present findings, it is suggested that exosomes serve as critical messengers in psoriatic inflammatory reactions.

A prior investigation by van Meteren et al. (2019) established that BaP enhances exosome release and alters exosomal protein markers in hepatocytes, indicating its impact on exosome biogenesis. In alignment with this, our study reveals heightened expression of ESCRT machinery proteins, Tsg101 and Alix, alongside diminished levels of tetraspanin CD63 in exosomes upon exposure to BaP, consistent with the findings of van Meteren et al. (2019). Moreover, the alterations observed in exosome marker protein expression in BaP-stimulated PBMCs compared to healthy controls mirror those seen in hepatocytes. Notably, our results highlight an escalated exosome release in response to BaP (refer to Supplementary Table S1), corroborating previous research findings. These collective outcomes imply that the influence of BaP on exosome-specific marker proteins involved in biosynthesis extends beyond hepatocytes, suggesting a potential role in the modulation of exosome biosynthesis in inflammatory diseases like psoriasis. Specifically, changes in the expression of exosomal marker proteins in psoriasis or under BaP stimulation could serve as promising biomarkers. Further research is imperative to validate these findings.

It has been established that secretions from cells in the cellular microenvironment can influence various cellular processes, including those involving exosomes. In addition to assessing the intended impact on the target exosomes, it is essential to consider the relevance of exosome-mediated effects in relation to the presence of compounds in the media. Notably, commonly used bovine serum as a medium supplement contains bovine RNA in extracellular vesicles and non-vesicular entities. These extracellular RNAs (exRNAs) have the potential to introduce confounding factors in molecular and functional readouts in recipient cells exposed to exosomes. In addition, it is crucial to consider factors secreted by exosome-independent processes. It can be postulated that autocrine substances, such as cell metabolites present in conditioned medium, may also play a role. These substances are produced directly by cells, independently of exosomes.

This study has several limitations. Although the original experiment design overlooked this aspect, it is necessary to broaden the scope of the PBMC experiments by including the exposure of psoriasis exosomes to psoriasis recipients as a control. BaP is known to induce apoptosis in HaCaT cells (Stolpmann et al., 2012), and it is plausible that apoptotic vesicles could potentially contaminate exosome preparations. Moreover, to unequivocally confirm that the observed effect is mediated by exosomes, additional investigations should involve co-cultures with donor and recipient PBMCs, as well as incubating the supernatant of donor PBMCs with recipient PBMCs. Additionally, the impact of BaP on psoriasis warrants confirmation through *in vivo* studies. Consequently, ongoing research employs an imiquimod (IMQ)-induced psoriasis mouse model to evaluate the *in vitro* findings regarding the effects of BaP.

In conclusion, this study pioneers the revelation that TCDD and BaP exert discernible effects on exosomes within human keratinocytes and PBMCs. Consequently, they fuel psoriatic inflammatory reactions by augmenting the expression of pro-inflammatory cytokines/chemokines and promoting Th17/Th1 cell differentiation in recipient cells. Our findings strongly imply the pivotal role of exosomes in exacerbating inflammatory skin responses to environmental toxicants. These results offer valuable insights into the involvement of exosomes in the pathogenesis of inflammatory cutaneous reactions induced by toxicants.

## Data Availability

The raw data supporting the conclusions of this article will be made available by the authors, without undue reservation.

## References

[B1] Alalaiwe A. Lin Y. K. Lin C. H. Wang P. W. Lin J. Y. Fang J. Y. (2020). The absorption of polycyclic aromatic hydrocarbons into the skin to elicit cutaneous inflammation: the establishment of structure-permeation and *in silico*-*in vitro*-*in vivo* relationships. Chemosphere 255, 126955. 10.1016/j.chemosphere.2020.126955 32416390

[B2] Antignac J. P. Marchand P. Gade C. Matayron G. Qannari El M. Le Bizec B. (2006). Studying variations in the PCDD/PCDF profile across various food products using multivariate statistical analysis. Anal. Bioanal. Chem. 384, 271–279. 10.1007/s00216-005-0129-z 16328251

[B3] Anttila H. S. Reitamo S. Erkko P. Ceska M. Moser B. Baggiolini M. (1992). Interleukin-8 immunoreactivity in the skin of healthy subjects and patients with palmoplantar pustulosis and psoriasis. J. Invest. Dermatol 98, 96–101. 10.1111/1523-1747.ep12495817 1728643

[B4] Chen S. C. Liao C. M. (2006). Health risk assessment on human exposed to environmental polycyclic aromatic hydrocarbons pollution sources. Sci. Total Environ. 366, 112–123. 10.1016/j.scitotenv.2005.08.047 16307791

[B5] Cheung K. L. Jarrett R. Subramaniam S. Salimi M. Gutowska-Owsiak D. Chen Y. L. (2016). Psoriatic T cells recognize neolipid antigens generated by mast cell phospholipase delivered by exosomes and presented by CD1a. J. Exp. Med. 213, 2399–2412. 10.1084/jem.20160258 27670592 PMC5068234

[B6] Di Meglio P. Duarte J. H. Ahlfors H. Owens N. D. Li Y. Villanova F. (2014). Activation of the aryl hydrocarbon receptor dampens the severity of inflammatory skin conditions. Immunity 40, 989–1001. 10.1016/j.immuni.2014.04.019 24909886 PMC4067745

[B7] Duan H. Koga T. Kohda F. Hara H. Urabe K. Furue M. (2001). Interleukin-8-positive neutrophils in psoriasis. J. Dermatol Sci. 26, 119–124. 10.1016/s0923-1811(00)00167-5 11378328

[B8] Duarte J. H. Di Meglio P. Hirota K. Ahlfors H. Stockinger B. (2013). Differential influences of the aryl hydrocarbon receptor on Th17 mediated responses *in vitro* and *in vivo* . PLoS One 8, e79819. 10.1371/journal.pone.0079819 24244565 PMC3828240

[B9] Esser C. Bargen I. Weighardt H. Haarmann-Stemmann T. Krutmann J. (2013). Functions of the aryl hydrocarbon receptor in the skin. Semin. Immunopathol. 35, 677–691. 10.1007/s00281-013-0394-4 23949496

[B10] Fernandez-Gallego N. Sanchez-Madrid F. Cibrian D. (2021). Role of AHR ligands in skin homeostasis and cutaneous inflammation. Cells 10, 3176. 10.3390/cells10113176 34831399 PMC8622815

[B11] Fortes C. Mastroeni S. Leffondré K. Sampogna F. Melchi F. Mazzotti E. (2005). Relationship between smoking and the clinical severity of psoriasis. Arch. Dermatol 141, 1580–1584. 10.1001/archderm.141.12.1580 16365261

[B12] Furue M. (2020). Regulation of skin barrier function via competition between AHR Axis versus IL-13/IL-4‒JAK‒STAT6/STAT3 Axis: pathogenic and therapeutic implications in atopic dermatitis. J. Clin. Med. 9, 3741. 10.3390/jcm9113741 33233866 PMC7700181

[B13] Griffiths C. E. M. Armstrong A. W. Gudjonsson J. E. Barker J. (2021). Psoriasis. Lancet 397, 1301–1315. 10.1016/S0140-6736(20)32549-6 33812489

[B14] Gutierrez-Vazquez C. Quintana F. J. (2018). Regulation of the immune response by the aryl hydrocarbon receptor. Immunity 48, 19–33. 10.1016/j.immuni.2017.12.012 29343438 PMC5777317

[B15] Harischandra D. S. Ghaisas S. Rokad D. Kanthasamy A. G. (2017). Exosomes in toxicology: relevance to chemical exposure and pathogenesis of environmentally linked diseases. Toxicol. Sci. 158, 3–13. 10.1093/toxsci/kfx074 28505322 PMC5837599

[B16] Hong C. H. Lee C. H. Yu H. S. Huang S. K. (2016). Benzopyrene, a major polyaromatic hydrocarbon in smoke fume, mobilizes Langerhans cells and polarizes Th2/17 responses in epicutaneous protein sensitization through the aryl hydrocarbon receptor. Int. Immunopharmacol. 36, 111–117. 10.1016/j.intimp.2016.04.017 27129092

[B17] Hotchkiss A. K. Rider C. V. Blystone C. R. Wilson V. S. Hartig P. C. Ankley G. T. (2008). Fifteen years after "Wingspread"--environmental endocrine disrupters and human and wildlife health: where we are today and where we need to go. Toxicol. Sci. 105, 235–259. 10.1093/toxsci/kfn030 18281716 PMC2721670

[B18] Jankovic S. Raznatovic M. Marinkovic J. Jankovic J. Maksimovic N. (2009). Risk factors for psoriasis: a case-control study. J. Dermatol 36, 328–334. 10.1111/j.1346-8138.2009.00648.x 19500181

[B19] Jiang M. Fang H. Shao S. Dang E. Zhang J. Qiao P. (2019). Keratinocyte exosomes activate neutrophils and enhance skin inflammation in psoriasis. FASEB J. 33, 13241–13253. 10.1096/fj.201900642R 31539277

[B20] Kalluri R. Lebleu V. S. (2020). The biology, function, and biomedical applications of exosomes. Science 367, eaau6977. 10.1126/science.aau6977 32029601 PMC7717626

[B21] Kamal A. Cincinelli A. Martellini T. Malik R. N. (2015). A review of PAH exposure from the combustion of biomass fuel and their less surveyed effect on the blood parameters. Environ. Sci. Pollut. Res. Int. 22, 4076–4098. 10.1007/s11356-014-3748-0 25410307

[B22] Kim H. O. Kim J. H. Chung B. Y. Choi M. G. Park C. W. (2014). Increased expression of the aryl hydrocarbon receptor in patients with chronic inflammatory skin diseases. Exp. Dermatol 23, 278–281. 10.1111/exd.12350 24521260

[B23] Kim H. R. Kang S. Y. Kim H. O. Park C. W. Chung B. Y. (2020). Role of aryl hydrocarbon receptor activation and autophagy in psoriasis-related inflammation. Int. J. Mol. Sci. 21, 2195. 10.3390/ijms21062195 32235789 PMC7139675

[B24] Kim H. R. Kim J. C. Kang S. Y. Kim H. O. Park C. W. Chung B. Y. (2021). Rapamycin alleviates 2,3,7,8-Tetrachlorodibenzo-p-dioxin-Induced aggravated dermatitis in mice with imiquimod-induced psoriasis-like dermatitis by inducing autophagy. Int. J. Mol. Sci. 22, 3968. 10.3390/ijms22083968 33921372 PMC8069848

[B25] Ko H. P. Okino S. T. Ma Q. Whitlock J. P. Jr. (1996). Dioxin-induced CYP1A1 transcription *in vivo*: the aromatic hydrocarbon receptor mediates transactivation, enhancer-promoter communication, and changes in chromatin structure. Mol. Cell Biol. 16, 430–436. 10.1128/mcb.16.1.430 8524325 PMC231019

[B26] Lebwohl M. G. Stein Gold L. Strober B. Papp K. A. Armstrong A. W. Bagel J. (2021). Phase 3 trials of tapinarof cream for plaque psoriasis. N. Engl. J. Med. 385, 2219–2229. 10.1056/NEJMoa2103629 34879448

[B27] Lowes M. A. Kikuchi T. Fuentes-Duculan J. Cardinale I. Zaba L. C. Haider A. S. (2008). Psoriasis vulgaris lesions contain discrete populations of Th1 and Th17 T cells. J. Invest. Dermatol 128, 1207–1211. 10.1038/sj.jid.5701213 18200064

[B28] Mandal P. K. (2005). Dioxin: a review of its environmental effects and its aryl hydrocarbon receptor biology. J. Comp. Physiol. B 175, 221–230. 10.1007/s00360-005-0483-3 15900503

[B29] Ni X. Lai Y. (2020). Keratinocyte: a trigger or an executor of psoriasis? J. Leukoc. Biol. 108, 485–491. 10.1002/JLB.5MR0120-439R 32170886

[B30] Quintana F. J. Basso A. S. Iglesias A. H. Korn T. Farez M. F. Bettelli E. (2008). Control of T(reg) and T(H)17 cell differentiation by the aryl hydrocarbon receptor. Nature 453, 65–71. 10.1038/nature06880 18362915

[B31] Reyes H. Reisz-Porszasz S. Hankinson O. (1992). Identification of the Ah receptor nuclear translocator protein (Arnt) as a component of the DNA binding form of the Ah receptor. Science 256, 1193–1195. 10.1126/science.256.5060.1193 1317062

[B32] Rothhammer V. Quintana F. J. (2019). The aryl hydrocarbon receptor: an environmental sensor integrating immune responses in health and disease. Nat. Rev. Immunol. 19, 184–197. 10.1038/s41577-019-0125-8 30718831

[B33] Rudyak S. G. Usakin L. A. Tverye E. A. Orekhov A. S. Belushkina N. N. Paus R. (2018). Retinoic acid co-treatment aggravates severity of dioxin-induced skin lesions in hairless mice via induction of inflammatory response. Biochem. Biophys. Res. Commun. 506, 854–861. 10.1016/j.bbrc.2018.10.126 30389142

[B34] Schecter A. Gasiewicz T. A. (2003). Dioxins and health. Hoboken, NJ: John Wiley and Sons Inc.

[B35] Shao S. Fang H. Zhang J. Jiang M. Xue K. Ma J. (2019). Neutrophil exosomes enhance the skin autoinflammation in generalized pustular psoriasis via activating keratinocytes. FASEB J. 33, 6813–6828. 10.1096/fj.201802090RR 30811955

[B36] Smith K. J. Boyer J. A. Muku G. E. Murray I. A. Gowda K. Desai D. (2017a). Editor's highlight: ah receptor activation potentiates neutrophil chemoattractant (C-X-C motif) ligand 5 expression in keratinocytes and skin. Toxicol. Sci. 160, 83–94. 10.1093/toxsci/kfx160 28973351 PMC5837612

[B37] Smith S. H. Jayawickreme C. Rickard D. J. Nicodeme E. Bui T. Simmons C. (2017b). Tapinarof is a natural AhR agonist that resolves skin inflammation in mice and humans. J. Invest. Dermatol 137, 2110–2119. 10.1016/j.jid.2017.05.004 28595996

[B38] Stolpmann K. Brinkmann J. Salzmann S. Genkinger D. Fritsche E. Hutzler C. (2012). Activation of the aryl hydrocarbon receptor sensitises human keratinocytes for CD95L- and TRAIL-induced apoptosis. Cell death Dis. 3 (9), e388. 10.1038/cddis.2012.127 22951985 PMC3461363

[B39] Strober B. Stein Gold L. Bissonnette R. Armstrong A. W. Kircik L. Tyring S. K. (2022). One-year safety and efficacy of tapinarof cream for the treatment of plaque psoriasis: results from the PSOARING 3 trial. J. Am. Acad. Dermatol 87, 800–806. 10.1016/j.jaad.2022.06.1171 35772599

[B40] Tang H. Jin X. Li Y. Jiang H. Tang X. Yang X. (2014). A large-scale screen for coding variants predisposing to psoriasis. Nat. Genet. 46, 45–50. 10.1038/ng.2827 24212883

[B41] Than U. T. T. Leavesley D. I. Parker T. J. (2019). Characteristics and roles of extracellular vesicles released by epidermal keratinocytes. J. Eur. Acad. Dermatol Venereol. 33, 2264–2272. 10.1111/jdv.15859 31403744

[B42] Tsoi L. C. Stuart P. E. Tian C. Gudjonsson J. E. Das S. Zawistowski M. (2017). Large scale meta-analysis characterizes genetic architecture for common psoriasis associated variants. Nat. Commun. 8, 15382. 10.1038/ncomms15382 28537254 PMC5458077

[B43] Tsuji G. Takahara M. Uchi H. Takeuchi S. Mitoma C. Moroi Y. (2011). An environmental contaminant, benzo(a)pyrene, induces oxidative stress-mediated interleukin-8 production in human keratinocytes via the aryl hydrocarbon receptor signaling pathway. J. Dermatol Sci. 62, 42–49. 10.1016/j.jdermsci.2010.10.017 21316925

[B44] Van Meteren N. Lagadic-Gossmann D. Chevanne M. Gallais I. Gobart D. Burel A. (2019). Polycyclic aromatic hydrocarbons can trigger hepatocyte release of extracellular vesicles by various mechanisms of action depending on their affinity for the aryl hydrocarbon receptor. Toxicol. Sci. 171, 443–462. 10.1093/toxsci/kfz157 31368503

[B45] Xiong M. Zhang Q. Hu W. Zhao C. Lv W. Yi Y. (2021). The novel mechanisms and applications of exosomes in dermatology and cutaneous medical aesthetics. Pharmacol. Res. 166, 105490. 10.1016/j.phrs.2021.105490 33582246

[B46] Xunian Z. Kalluri R. (2020). Biology and therapeutic potential of mesenchymal stem cell-derived exosomes. Cancer Sci. 111, 3100–3110. 10.1111/cas.14563 32639675 PMC7469857

