# Corrigendum: Exosomes released by environmental pollutant-stimulated Keratinocytes/PBMCs can trigger psoriatic inflammation in recipient cells via the AhR signaling pathway

**DOI:** 10.3389/fmolb.2024.1494968

**Published:** 2024-10-23

**Authors:** Hye Ran Kim, So Yeon Lee, Ga Eun You, Chun Wook Park, Hye One Kim, Bo Young Chung

**Affiliations:** ^1^ Department of Dermatology, Kangnam Sacred Heart Hospital, Hallym University College of Medicine, Seoul, Republic of Korea; ^2^ Research and Development Institute, Biosolution, Seoul, Republic of Korea

**Keywords:** exosomes, benzo[a]pyrene, aryl hydrocarbon receptor, psoriasis, 2,3,7,8-tetrachlorodibenzo-p-dioxin (TCDD)

In the published article, there was an error.

A correction has been made to the **Introduction**, Paragraph 4. This sentence previously stated:

“While the impact of environmental pollutants on exosomes has been explored in neurodegenerative diseases, several cancers, and lung and liver diseases (Harischandra et al., 2017; van Meteren et al., 2019). Is available concerning their effects on skin diseases.”

The corrected sentence appears below:

“While the impact of environmental pollutants on exosomes has been explored in neurodegenerative diseases, several cancers, and lung and liver diseases (Harischandra et al., 2017; van Meteren et al., 2019), nothing is available concerning their effects on skin diseases.”

In the published article, there was an error in Figure 3 as published. In Figure 3A, the third graph of the relative intensity of Western blot bands was incorrectly labeled “P-PEK” instead of “P-ERK”. The corrected Figure 3 and its caption appear below.

**FIGURE 3:**
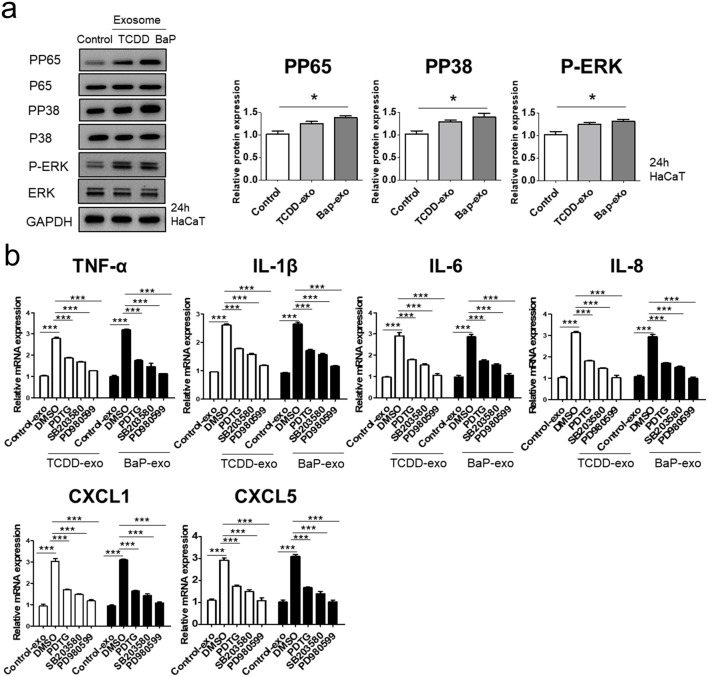
The effects of exosomes derived from BaP- or TCDD-treated HaCaT cells on the p65/NF-κB, p38/MAPK and ERK/MAPK signaling pathways in recipient HaCaT cells. **(A)** P-P65, P-P38 and P-ERK expression treated with exosomes derived from BaP- or TCDD-treated HaCaT cells by Western blotting. The relative expression was normalized to GAPDH (*n* = 3). The relative intensity of each band was quantified by densitometric scan. **(B)** TNF-α, IL-1β, IL-6, IL-8, CXCL1, and CXCL5 expression with the exosome-derived from BaP- or TCDD-treated HaCaT cell in the absence or presence of inhibitors of P65 (PDTC, 10 µM), P38 (SB203580, 10 µM), or ERK (PD980599, 5 µM) by quantitative PCR. Statistics: mean ± S.D. (*n* = 3). Statistical significance was determined by one-way ANOVA followed by Tukey’s multiple comparison test. ^**^ *p* < 0.01 and ^***^ *p* < 0.001. PDTC, pyrrolidine dithiocarbamate, exo, exosome, BaP, benzo [a]pyrene.

In the published article, there was an error in Figure 7 as published. The color of some of the images was different from the one in the original figure. The corrected Figure 7 and its caption appear below.

**FIGURE 7:**
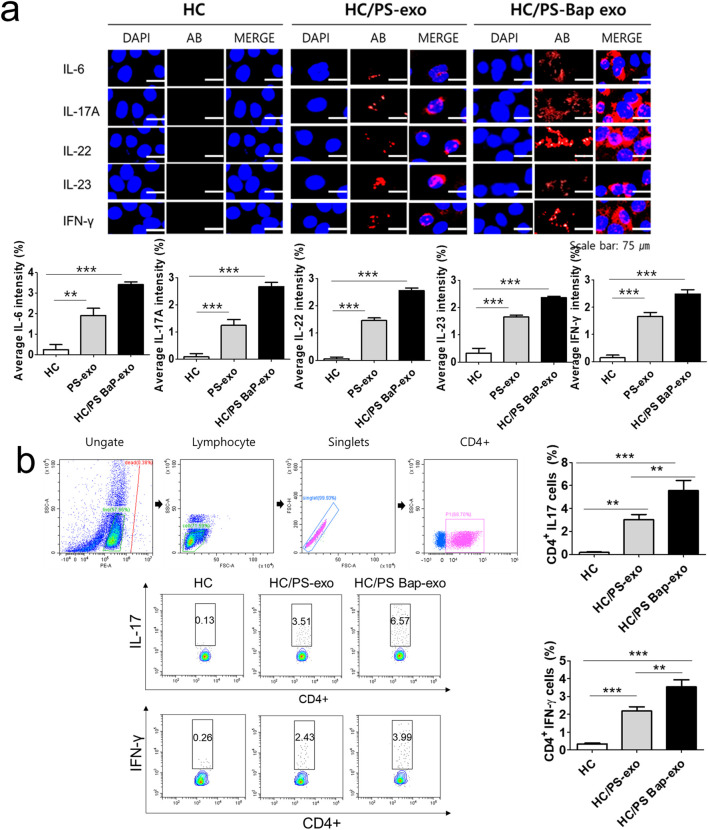
The effects of exosomes derived from BaP-treated PS PBMCs on proinflammatory cytokine expression, and the distribution of IL-17A-positive and IFN-γ-positive CD4^+^ T cells in recipient HC PBMCs. **(A)** The expressions of IL-6, IL-17A, IL-22, IL-23, and IFN-γ by immunofluorescence. Results are a representation of one sample of each group (PS = 3, HC = 3). Scale bar = 75 µm. The fluorescence intensity was semi-quantitatively analyzed and the results are presented as the mean optical density with standard deviation based on three different digital images. Statistical significance was determined by one-way ANOVA followed by Tukey’s multiple comparison test. ^***^ *p* < 0.001 **(B)** Representative dot plots and the percentages of IL-17A-positive and IFN-γ-positive CD4^+^ T cells. The percentage of IL-17A-positive and IFN-γ-positive CD4^+^ T cells by flow cytometric analysis. The percentage of dead cells in IL-17 and CD4 was 1.86% for HC, 1.09% for HC/PS-exo, and 0.21% for HC/PS Bap-exo. For INF-y and CD4, the percentages were 0.38% for both HC and HC/PS-exo, and 0.11% for HC/PS Bap-exo and 0.57%. Results are a representation of one sample of each group (PS = 3, HC = 3). Statistics: mean ± S.D. ^**^ *p* < 0.05 and ^***^ *p* < 0.001. exo, exosome, BaP, benzo [a]pyrene, PS, psoriasis patient, HC, healthy control.

The authors apologize for these errors and state that these do not change the scientific conclusions of the article in any way. The original article has been updated.

