# Supplementary figures and images for: Exosomes released by environmental pollutant-stimulated Keratinocytes/PBMCs can trigger psoriatic inflammation in recipient cells via the AhR signaling pathway
Source: Front Mol Biosci. 2024 Jan 15;10:1324692. doi: 10.3389/fmolb.2023.1324692 (PMC10822922; doi:10.3389/fmolb.2023.1324692)

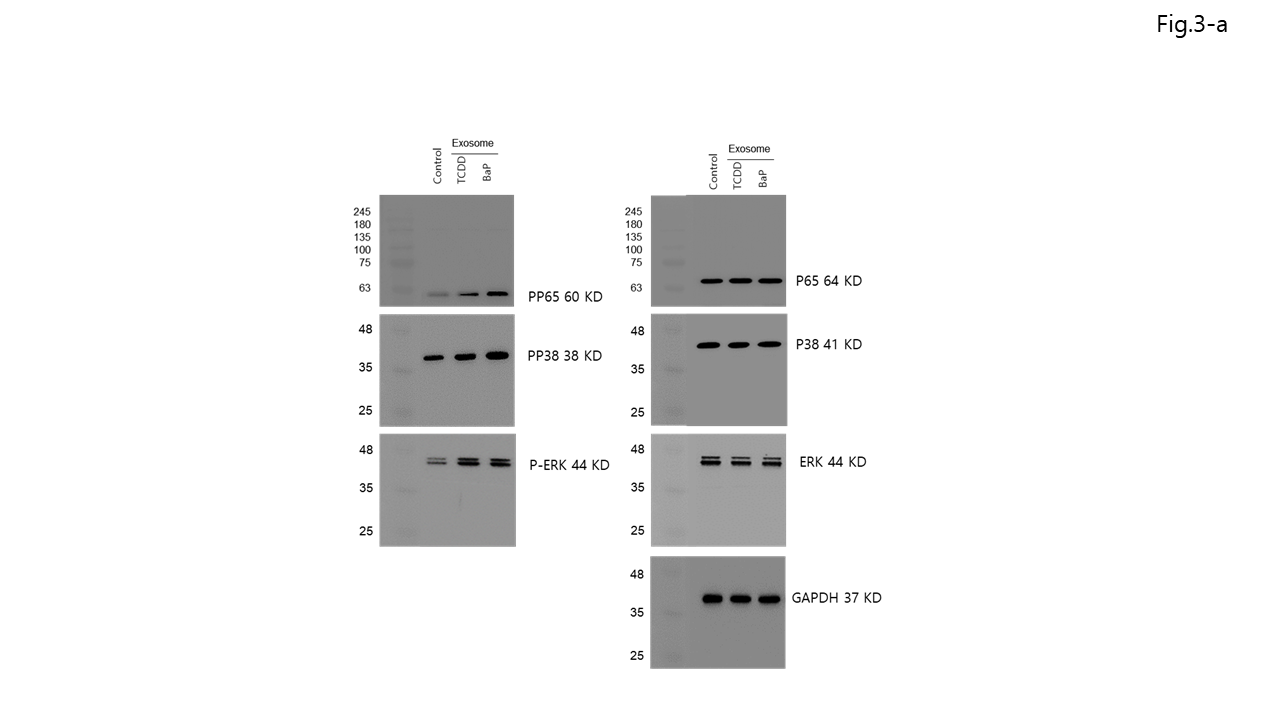

Supplement: Supplementary file 1 [file Image3.TIF]

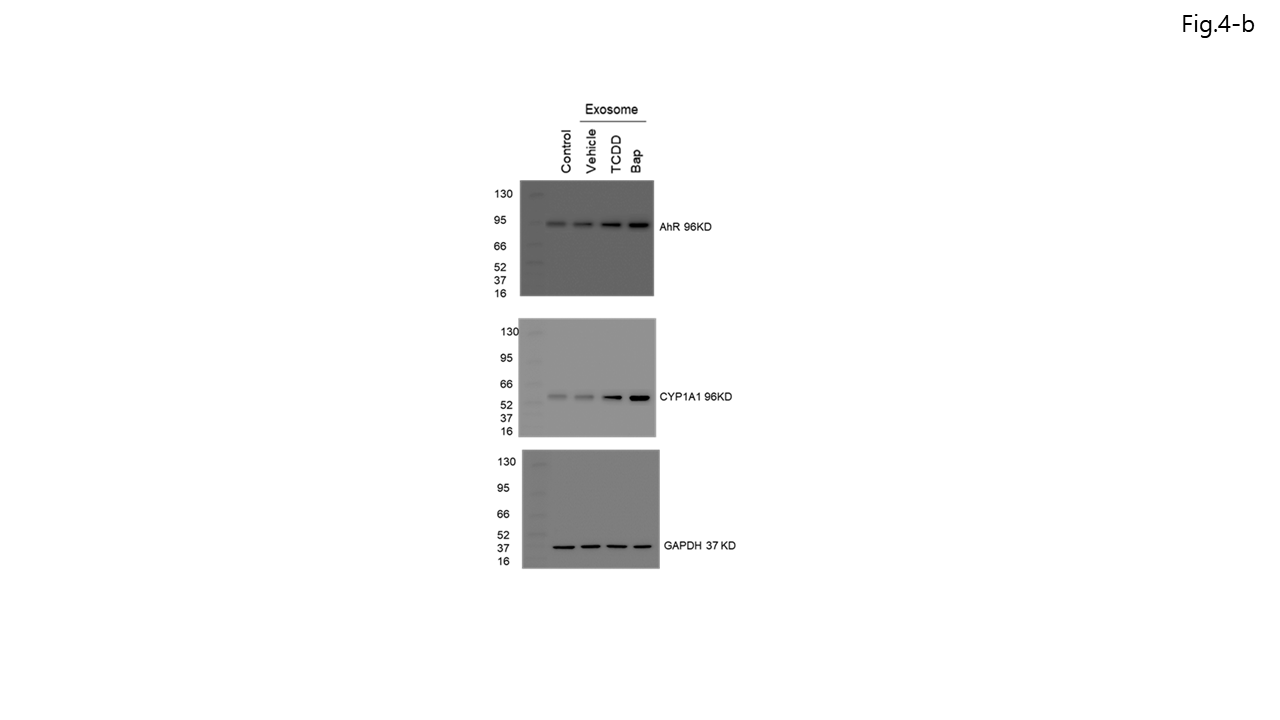

Supplement: Supplementary file 2 [file Image4.TIF]

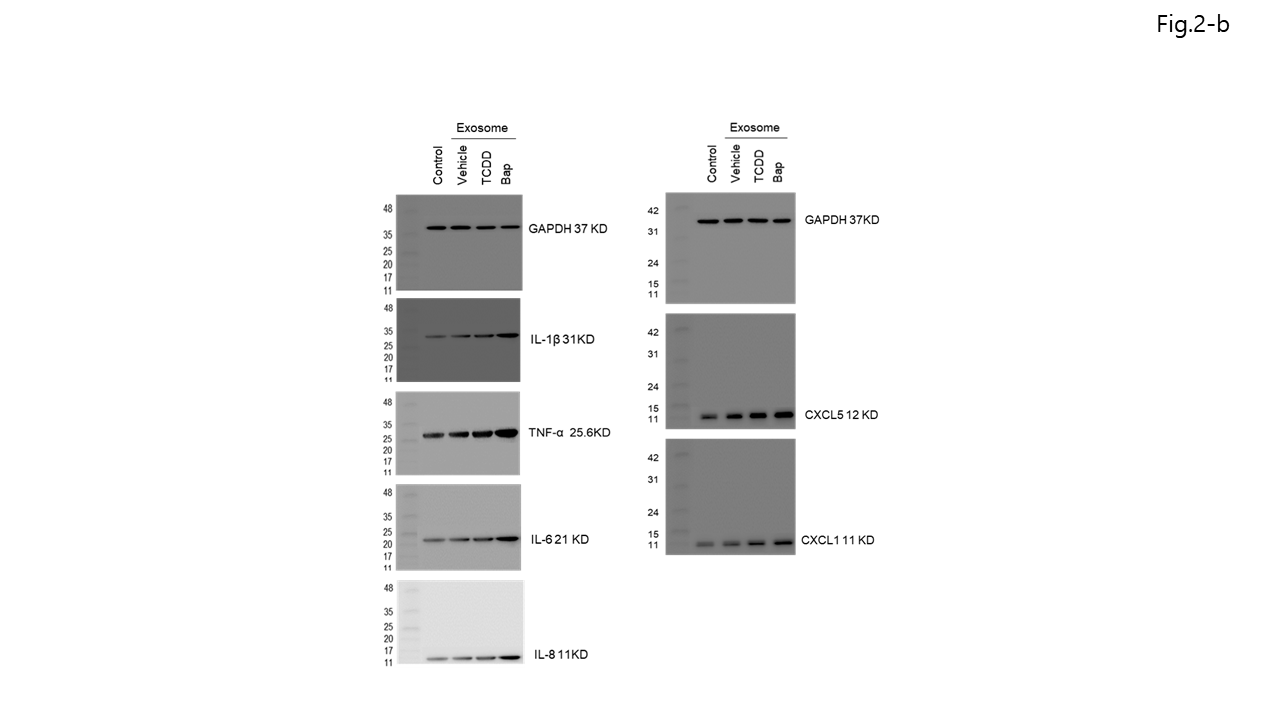

Supplement: Supplementary file 3 [file Image2.TIF]

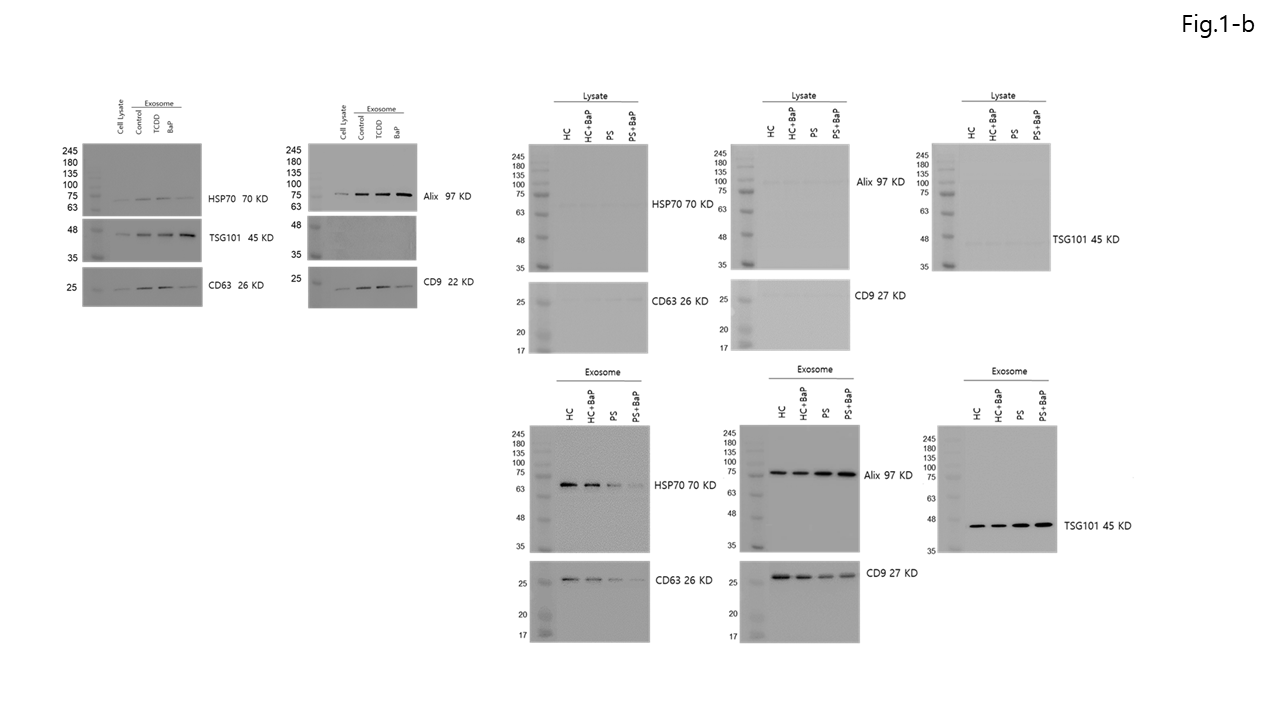

Supplement: Supplementary file 4 [file Image1.TIF]

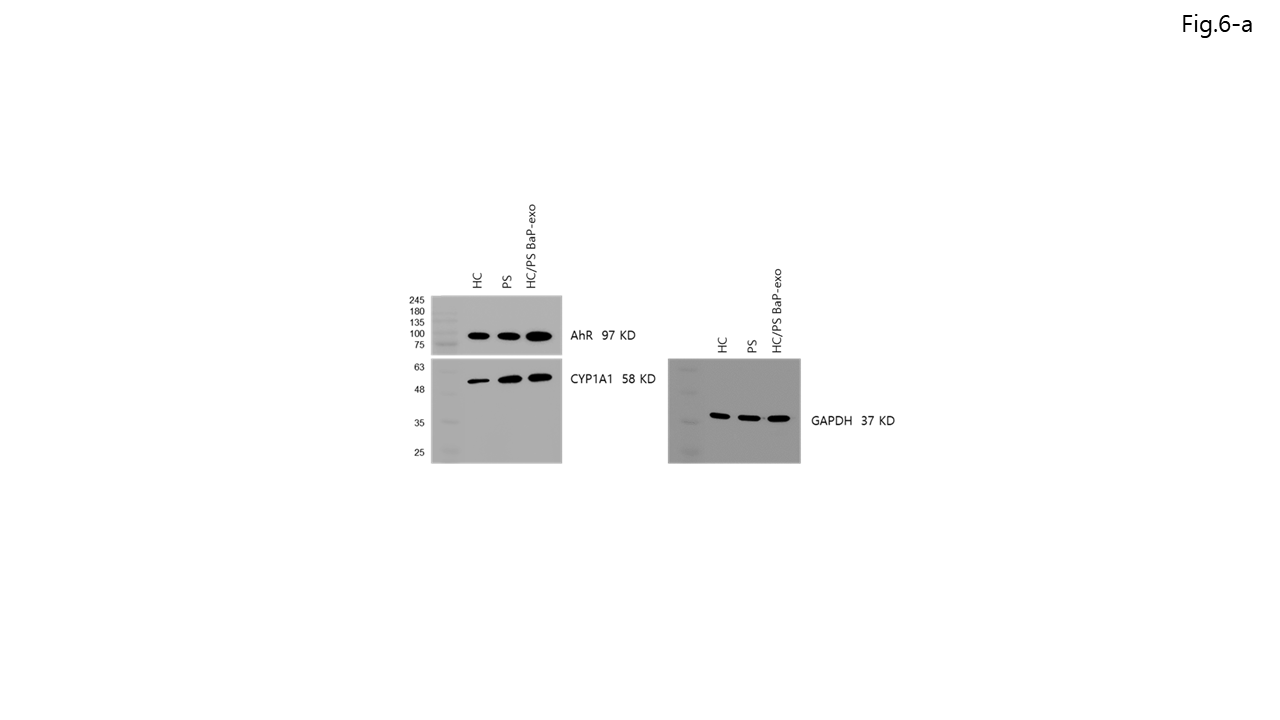

Supplement: Supplementary file 5 [file Image5.TIF]
