# Supplementary material for: Exosomes released by environmental pollutant-stimulated Keratinocytes/PBMCs can trigger psoriatic inflammation in recipient cells via the AhR signaling pathway
Source: Front Mol Biosci. 2024 Jan 15;10:1324692. doi: 10.3389/fmolb.2023.1324692 (PMC10822922; doi:10.3389/fmolb.2023.1324692)
Supplement: Supplementary file 6 [file DataSheet1.docx]

Supplementary Material

# Supplementary Figures and Tables

## Supplementary Figures


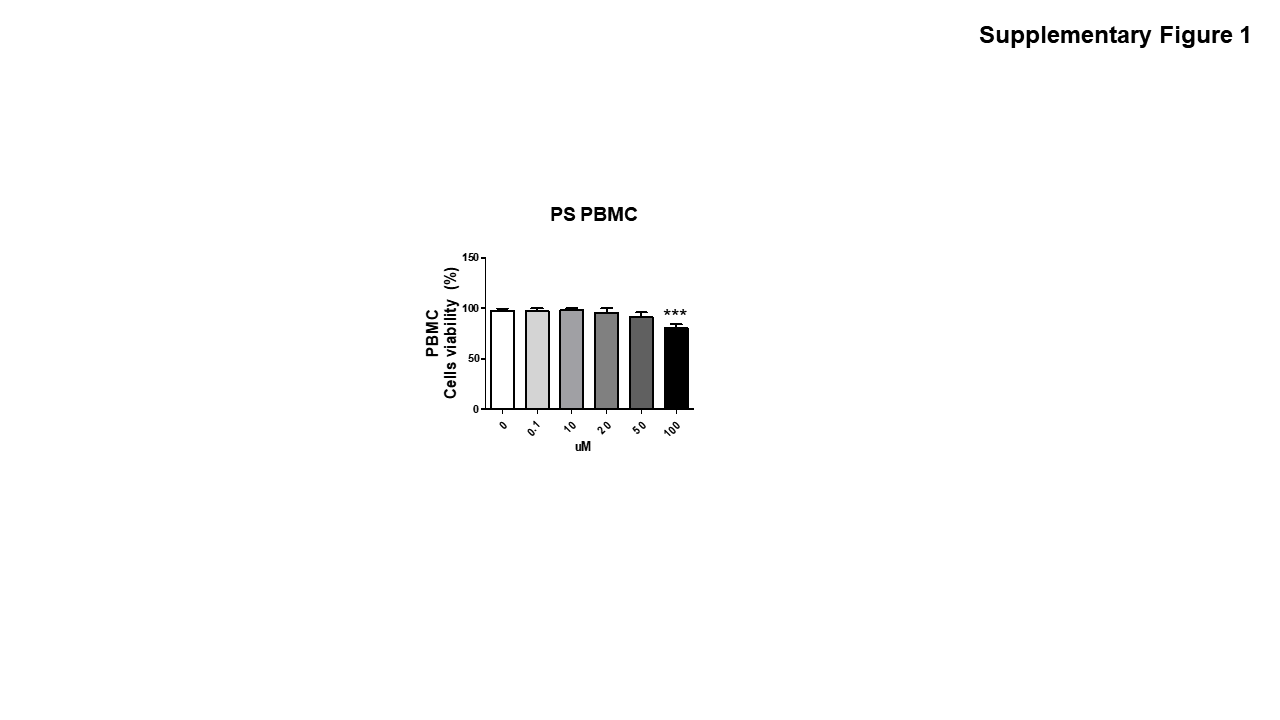


## Supplementary Figure 1. The effect of BaP on the viability of PBMCs from patients with psoriasis. After treatment of PBMCs from psoriasis patients with BaP (0, 0.1, 10, 20, 50 and 100 µM) for 24 h, cell viability was measured by 3-(4,5-dimethylthiazol-2yl)-2,5-diphenyl-tetrazolium bromide (MTT) assays. Data represent the mean ± S.D. of three independent experiments. Statistical significance was determined by one-way ANOVA followed by Tukey’s multiple comparison test. ***P <0.001.


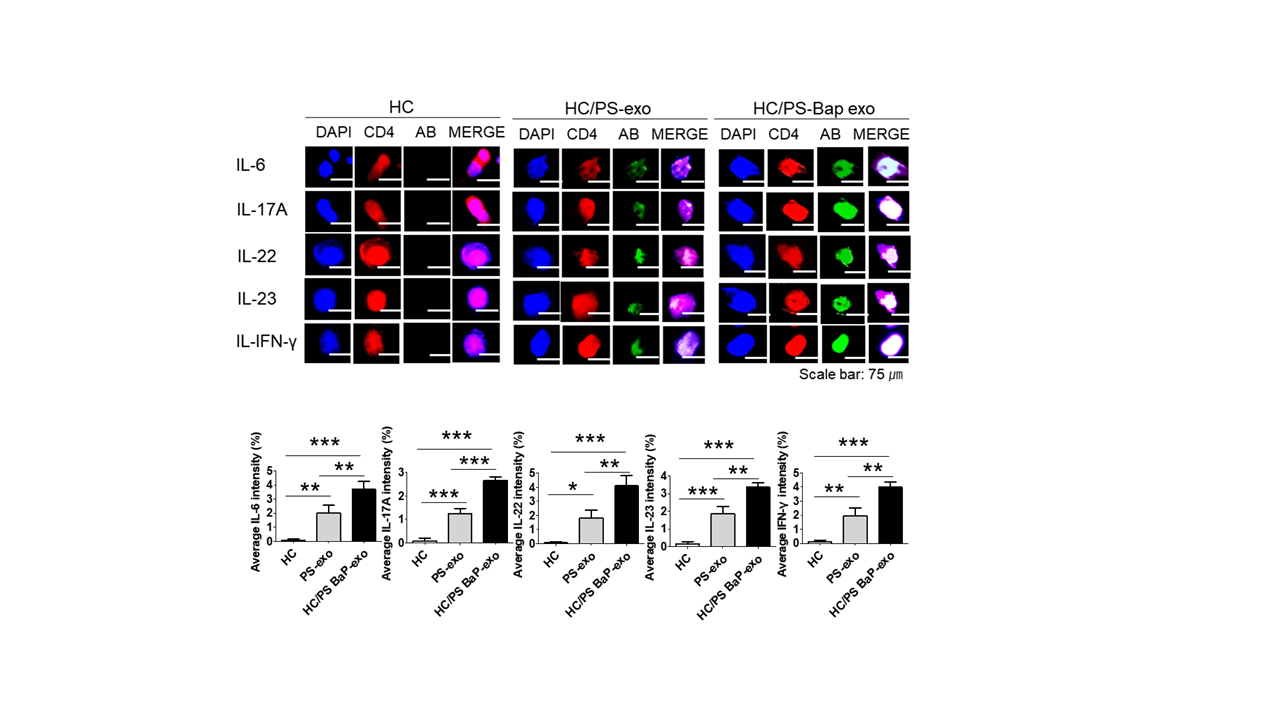


**Supplementary Figure 2.** The effects of exosomes derived from BaP-treated PS PBMCs on proinflammatory cytokine expression. The expressions of IL-6, IL-17A, IL-22, IL-23 and IFN-γ by immunofluorescence. Results are a representation of one sample of each group (PS=3, HC=3). DAPI (Abcam, Cambridge, UK) is used for nucleus staining (blue). The anti-CD4 antibody (Santa Cruz, Heidelberg, Germany) is employed for lymphocyte staining (red). The antibodies against IL6, IL17A, IL22, IL23, and IFN-γ, all sourced from Abcam in Cambridge, UK, are utilized for cytokine staining (green). Additionally, all secondary fluorescence is labeled with FITC. Scale bar = 75 µm. The fluorescence intensity was semi-quantitatively analyzed and the results are presented as the mean optical density with standard deviation based on three different digital images. Statistical significance was determined by one-way ANOVA followed by Tukey’s multiple comparison test. ***P <0.001 exo, exosome, BaP, benzo[a]pyrene, PS, psoriasis patient, HC, healthy control.


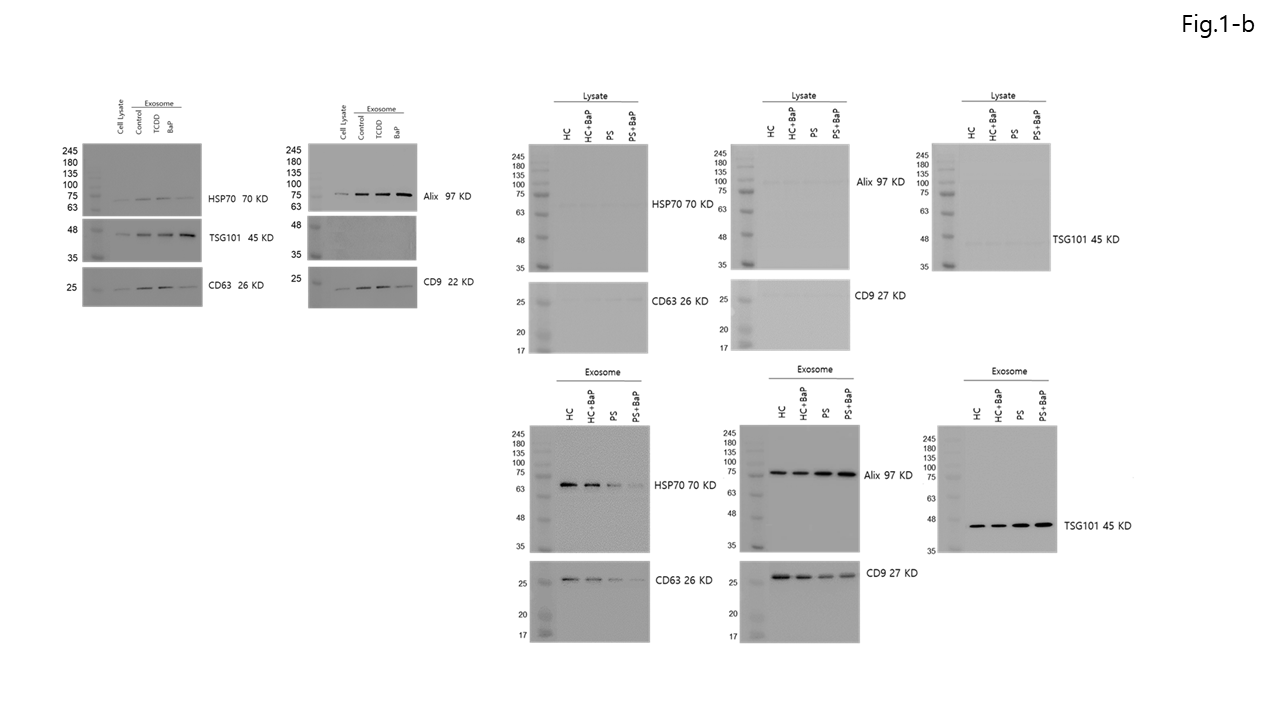

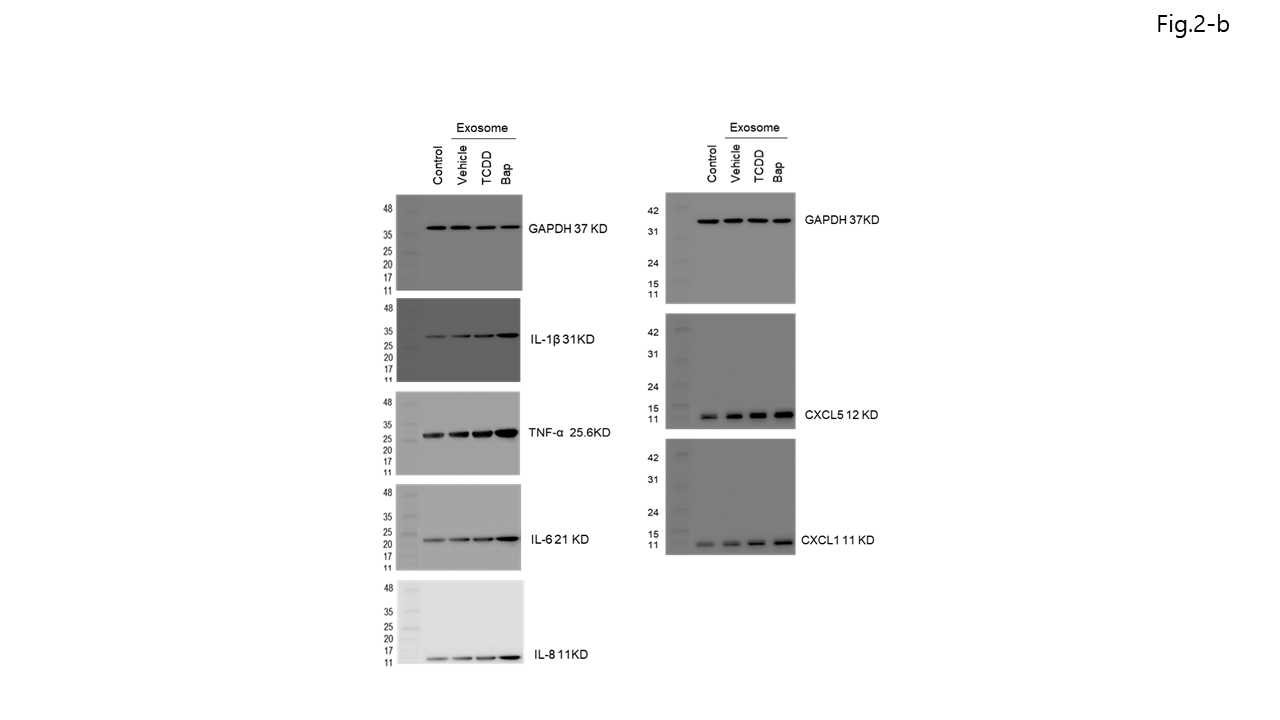


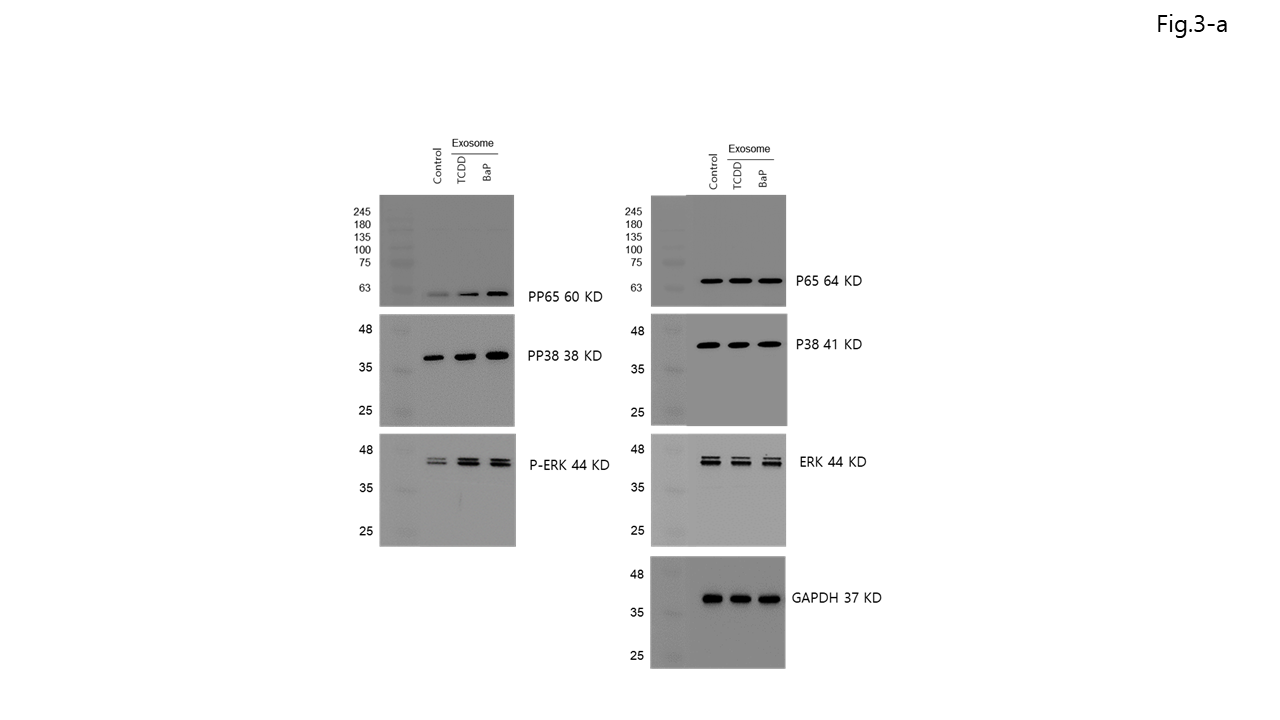


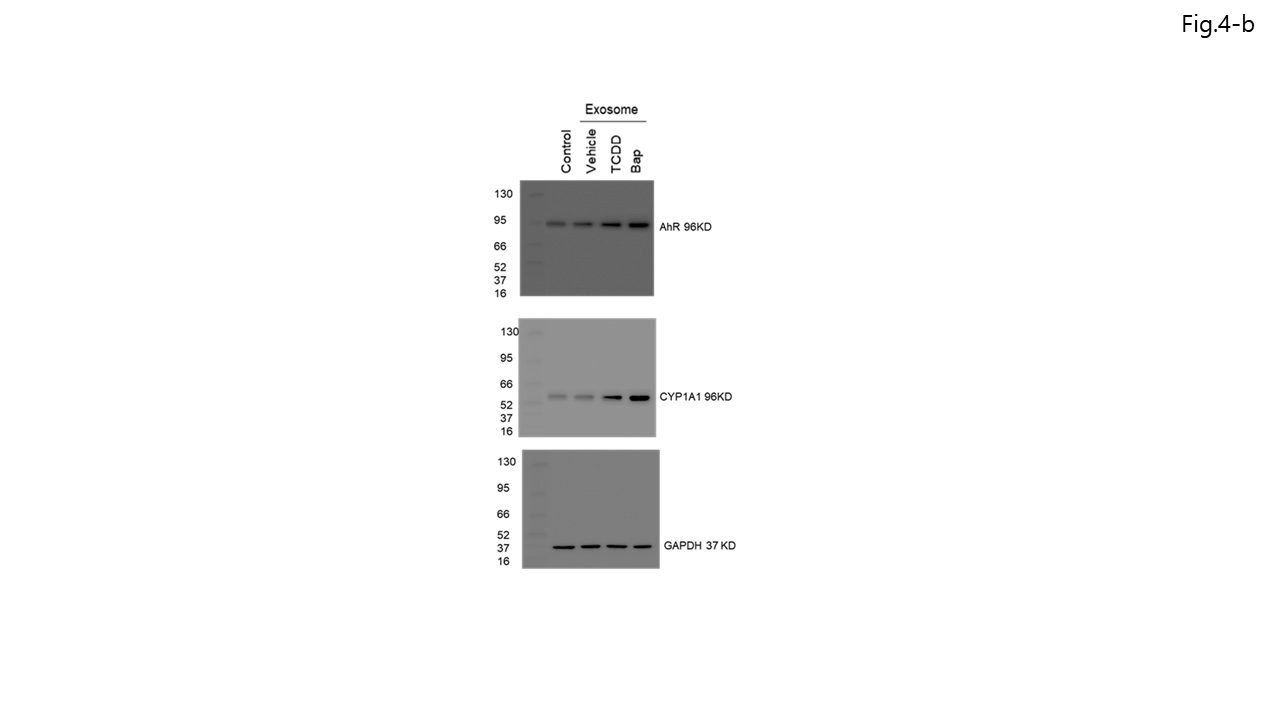


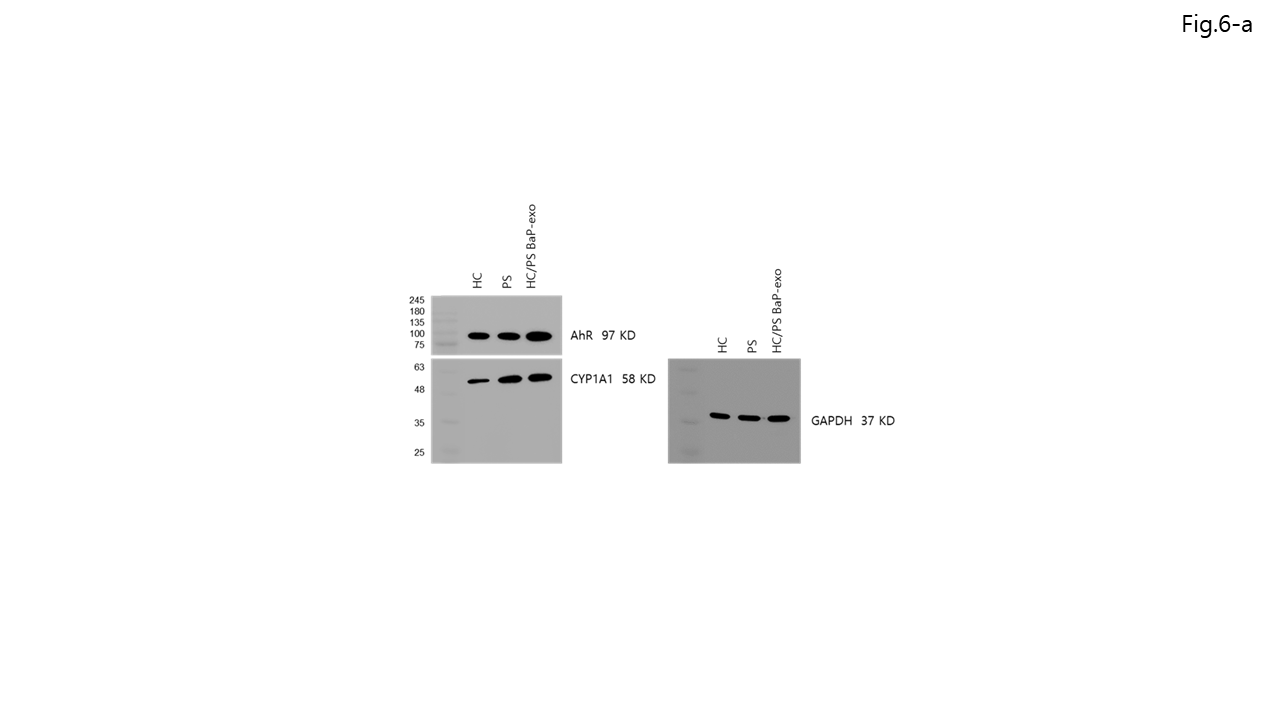


**Supplementary Figure 3.** Western blots in full with size standard.

## Supplementary Tables

| Group | Particles/mL |
| --- | --- |
| HaCaT exo | 6.14 x10^8^ |
| BaP-treated HaCaT exo | 7.01 x10^8^ |
| HC PBMC exo | 4.15 x10^8^ |
| BaP-treated HC PBMC exo | 6.95 x10^8^ |
| PS PBMC exo | 4.59 x10^8^ |
| BaP-treated PS PBMC exo | 8.11 x10^8^ |

**Supplementary Table 1.** The amount of exosome release. exo, exosome, BaP, benzo[a]pyrene, PS, psoriasis patient, HC, healthy control.
